# mDia1/3 generate cortical F-actin meshwork in Sertoli cells that is continuous with contractile F-actin bundles and indispensable for spermatogenesis and male fertility

**DOI:** 10.1371/journal.pbio.2004874

**Published:** 2018-09-26

**Authors:** Satoko Sakamoto, Dean Thumkeo, Hiroshi Ohta, Zhen Zhang, Shuangru Huang, Pakorn Kanchanawong, Takayoshi Fuu, Sadanori Watanabe, Kentaro Shimada, Yoshitaka Fujihara, Shosei Yoshida, Masahito Ikawa, Naoki Watanabe, Mitinori Saitou, Shuh Narumiya

**Affiliations:** 1 Medical Innovation Center, Kyoto University Graduate School of Medicine, Kyoto, Japan; 2 Department of Drug Discovery Medicine, Kyoto University Graduate School of Medicine, Kyoto, Japan; 3 Department of Anatomy and Cell Biology, Kyoto University Graduate School of Medicine, Kyoto, Japan; 4 Mechanobiology Institute, National University of Singapore, Singapore; 5 Department of Biomedical Engineering, National University of Singapore, Singapore; 6 Research Institute for Microbial Disease, Osaka University, Osaka, Japan; 7 National Institute for Basic Biology, Okazaki, Japan; 8 Department of Pharmacology, Kyoto University Graduate School of Medicine, Kyoto, Japan; 9 Laboratory of Single-Molecule Cell Biology, Kyoto University Graduate School of Biostudies, Kyoto, Japan; Cornell University, United States of America

## Abstract

Formin is one of the two major classes of actin binding proteins (ABPs) with nucleation and polymerization activity. However, despite advances in our understanding of its biochemical activity, whether and how formins generate specific architecture of the actin cytoskeleton and function in a physiological context in vivo remain largely obscure. It is also unknown how actin filaments generated by formins interact with other ABPs in the cell. Here, we combine genetic manipulation of formins mammalian diaphanous homolog1 (*mDia1*) and 3 (*mDia3*) with superresolution microscopy and single-molecule imaging, and show that the formins mDia1 and mDia3 are dominantly expressed in Sertoli cells of mouse seminiferous tubule and together generate a highly dynamic cortical filamentous actin (F-actin) meshwork that is continuous with the contractile actomyosin bundles. Loss of mDia1/3 impaired these F-actin architectures, induced ectopic noncontractile espin1-containing F-actin bundles, and disrupted Sertoli cell–germ cell interaction, resulting in impaired spermatogenesis. These results together demonstrate the previously unsuspected mDia-dependent regulatory mechanism of cortical F-actin that is indispensable for mammalian sperm development and male fertility.

## Author summary

Paternal genetic information is transmitted to the offspring via sperm. The unique cell morphology of the sperm plays essential roles in sperm transport through the female reproductive tract and in fertilization with oocytes. Sertoli cells are somatic cells located in the seminiferous tubules of the testis and are known to contribute to the development of sperm. While many studies have analyzed sperm development, the mechanisms underlying its morphogenesis remain obscure. In this work, we showed that the interaction between developing sperm and Sertoli cells is critical for sperm morphogenesis. We further unraveled that this interaction is strongly dependent on the cortical F-actin meshwork and contractile actomyosin bundles of Sertoli cells, and that two actin polymerization and nucleation factors of the formin family, mDia1 and mDia3, are involved in the generation of both actin-based structures. Loss of these formins in mice result in disrupted Sertoli cell actin structures, abnormal sperm morphology, and male infertility. We conclude that mDia1 and mDia3 play a role in sperm development through the regulation of the actin cytoskeletal architecture of Sertoli cells and that defects in these proteins might contribute to male infertility.

## Introduction

Filamentous actin (F-actin) nucleation and polymerization are controlled by actin nucleators, including actin-related protein2/3 (Arp2/3) and formin, in mammalian cells. Previous studies in typical cultured cell lines showed that Arp2/3 generates branched actin filaments, while formin generates straight actin filaments [1]. However, F-actin structure and dynamics regulated by formin in a variety of mammalian cell types and their physiological roles in vivo remain largely unknown. Mammalian diaphanous homolog (mDia) proteins, mammalian homologues of *Drosophila* diaphanous, belong to the formin family of proteins and consist of three isoforms in mammals, namely mDia1, mDia2, and mDia3 [2]. To unravel mDia-dependent F-actin structures in the mammalian body and explore their physiological functions in vivo, we generated mice deficient in each isoform and analyzed their phenotypes [3–5]. These studies showed the functional redundancy between mDia1 and mDia3 isoforms and demonstrated that mDia1/3-mediated F-actin is critical for neuroblast migration [5] and neuroepithelium integrity [6] in the developing brain, and mediates presynaptic plasticity of mature neurons in the adult brain [7]. However, the contribution and function of mDia-mediated F-actin in other systems remain an open question.

Spermatogenesis is a process by which spermatogonial stem cells give rise to spermatozoa through spermatocytes, round spermatids, and elongated spermatids in seminiferous tubules. Besides these germ cells, the seminiferous tubule contains a somatic constituent of the seminiferous epithelium, the Sertoli cells. Sertoli cells make adhesion with developing germ cells and provide them with structural and functional supports [8], which are indispensable for normal spermatogenesis. Adhesion between Sertoli cells and round spermatids is mediated by the adherens junction (AJ) [9]. In addition, a special form of cell–cell adhesion, the apical ectoplasmic specialization (ES) junction, is formed between Sertoli cells and spermatids in association with the elongation of the round spermatid [10,11]. In contrast to the AJ, which is a structure associated with contractile actomyosin bundles [12], the apical ES junction is typified by a layer of densely packed noncontractile F-actin bundles [13] concentrated immediately beneath the Sertoli cell plasma membrane. It is known that these noncontractile F-actin bundles are associated with actin bundling protein espin1 [14] and support elongated spermatid adhesion and orientation through the regulation of the apical ES junction [15,16]. Although the structure and function of AJ and ES junctions have been extensively investigated, how F-actin structures associated with these junctions in Sertoli cell are formed, interact with each other, and are maintained during spermatogenesis are largely obscure.

F-actin makes several different forms of architectures beneath the cell membrane, which are collectively termed cortical F-actin [17] and are determined by the environment surrounding the cell. For example, in cells cultured in suspension under steady state, F-actin in the cortex makes a highly dense and fine mesh structure with a homogeneous pore size of approximately 30 nm [18]. It was previously shown that the Arp2/3 complex dominantly nucleate these meshlike cortical actin filaments [18,19], although about 10% of the cortical F-actin of these cells was shown to be formin dependent [19]. On the other hand, cortical F-actin structures in adherent cells are more complicated because dynamic remodeling of cortical network results in additional F-actin architectures, such as the contractile stress fibers [20]. Stress fibers are mostly localized at the basal plane of the cell and are more visible than those finely organized filaments, making conventional imaging of cortical F-actin very challenging. Indeed, the structure and dynamics of cortical F-actin of the adherent cell remain largely unexplored to date, and how and which actin nucleators regulate them are unknown.

In this study, we show that both mDia1 and mDia3 are dominantly expressed in the Sertoli cells of mouse testes and are indispensable for sperm development and male fertility. Utilizing superresolution microscopy, we resolved the nanoscale F-actin architecture of Sertoli cells attached to the substrate and observed a previously unsuspected F-actin meshwork with a large mesh size of about 100 nm. Live imaging of F-actin has further revealed the highly dynamic nature of this cortical F-actin meshwork of the Sertoli cell. Moreover, genetic and pharmacological experiments have shown that this Sertoli cell cortical F-actin meshwork depends on actin nucleation and polymerization activity of formins, including mDia1/3, but not Arp2/3. Intriguingly, these mDia1/3-dependent actin filaments are required for the generation of contractile actomyosin bundles in Sertoli cells. Consequently, loss of mDia1/3 in Sertoli cells results in severe reduction of cortical F-actin meshwork and actomyosin bundles and, instead, unexpectedly causes ectopic formation of espin1-associated noncontractile F-actin bundles in the cell. Finally, we demonstrate that mDia-dependent cortical F-actin meshwork and contractile actomyosin in Sertoli cells are together critical for interaction with germ cells, which is required for their competency to support spermatogenesis.

## Results

### mDia1 and mDia3 are dominantly expressed in Sertoli cells and loss of mDia1/3 in Sertoli cells, but not germ cells, results in impaired spermatogenesis

When breeding mice in our colony, we found that, while *mDia1* knockout (KO) male mice [3] and *mDia3* KO male mice [5] were fully fertile, the mating of *mDia1/3* double knockout (DKO) male mice [10] yielded no offspring upon mating. The fertilization rate upon in vitro fertilization (IVF) of wild-type (WT) oocytes with *mDia1/3* DKO sperms was also greatly reduced, suggesting abnormality in the sperm (S1A and S1B Fig). Analysis of the epididymis of 8-wk-old male *mDia1/3* DKO mice further revealed reduced number, abnormal morphology, and impaired motility of *mDia1/3* DKO sperm compared with the control WT mice (S2A–S2I Fig). These results together suggest that the male infertility phenotype of *mDia1/3* DKO mice is likely due to impaired sperm development.

**Fig 1:**
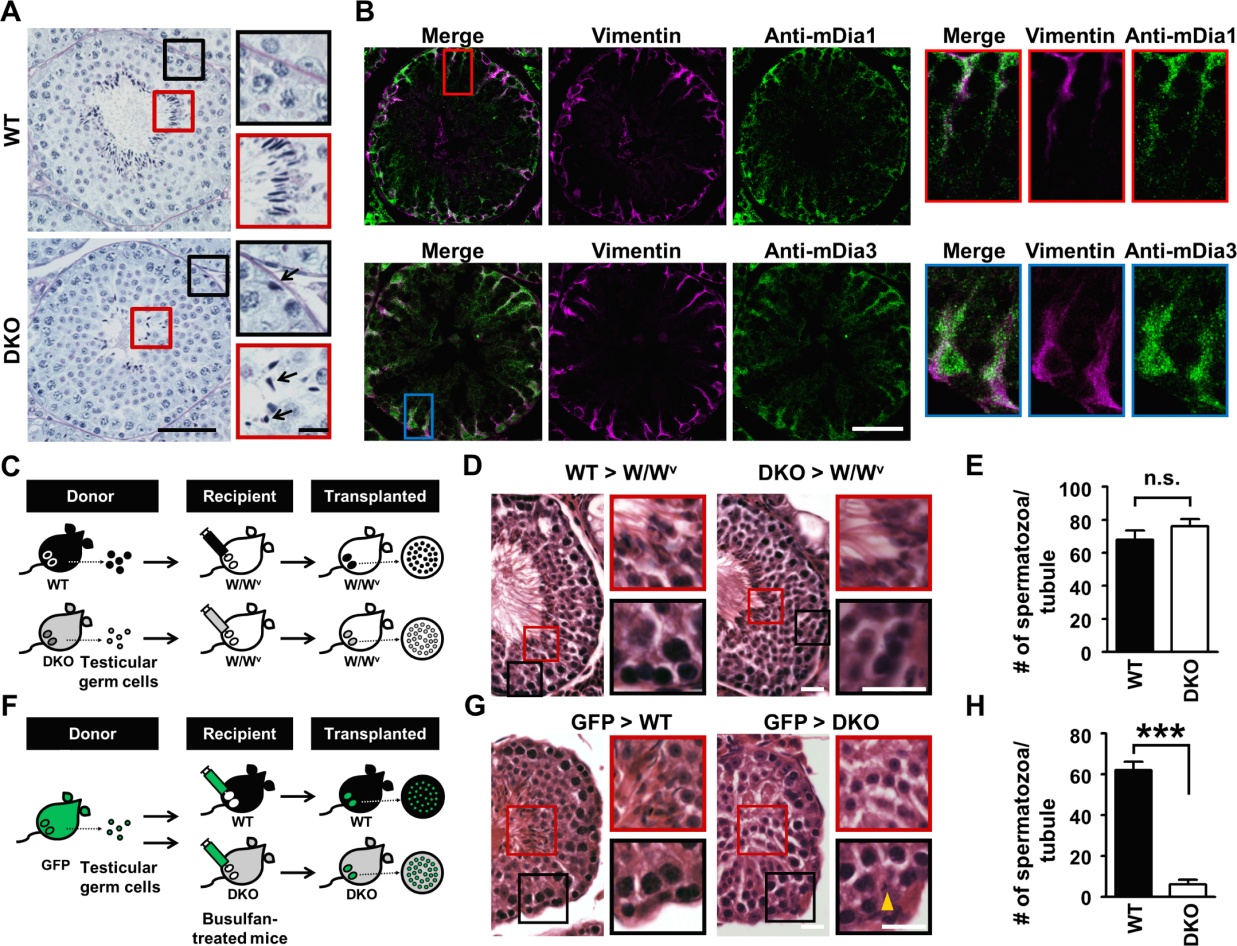
mDia1 and mDia3 are dominantly expressed in Sertoli cells and indispensable for spermatogenesis. (A) PAS-stained stage VIII seminiferous tubule sections from adult WT and *mDia1/3* DKO mice. The red (apical region) and black (basal region) boxes are magnified images of the corresponding boxed areas of the lower magnification. Note that in *mDia1/3* DKO mice, differentiated sperms are decreased in number and not properly orientated at the lumen of the seminiferous tubule (black arrows, red box), and elongated spermatids are ectopically localized proximal to the basal lamina (black arrow, black box). Scale bars, 50 μm (left) and 10 μm (right). (B) Immunofluorescence staining for mDia1 or mDia3 (green) and vimentin (magenta), a marker for Sertoli cells, in testis sections from WT adult mice. Note the strong mDia1 and mDia3 immunostaining signals in the cytoplasm of Sertoli cells. Scale bar, 100 μm. The red and blue boxes are magnified images of the corresponding boxed areas of the lower magnification. (C) Schematic representation of testicular germ cell suspensions from WT or *mDia1/3* DKO mice transplanted into the Sertoli cell-only testes of sterile *W/W*^*V*^ mutant mice. (D) HE-stained testis sections from *W/W*^*v*^ mice transplanted with WT or *mDia1/3* DKO testicular germ cell suspensions. Note that the histology of *W/W*^*v*^ mouse testes transplanted with *mDia1/3* DKO testicular cell suspensions exhibits apparently normal spermatogenesis. Scale bars, 25 μm. The red and black boxes are magnified images of the corresponding boxed areas of the lower magnification. (E) Quantification of the number of spermatozoa per seminiferous tubule in *W/W*^*v*^ mice transplanted with WT testicular germ cell suspensions (13 tubules of two mice) or *mDia1/3* DKO testicular germ cell suspensions (17 tubules of two mice). Data are represented as mean ± SEM. (F) Schematic representation of the transplantation of testicular germ cell suspensions from *acro/act-EGFP* mice into the busulfan-treated Sertoli cell-only testes of WT or *mDia1/3* DKO mice. (G) HE-stained testis sections from WT or *mDia1/3* DKO mice transplanted with *acro/act-EGFP* mice-derived testicular germ cell suspensions. Note that *mDia1/3* DKO mouse testis transplanted with *acro/act-EGFP* mice-derived testicular germ cell suspensions exhibits histologically abnormal spermatogenesis, similar to *mDia1/3* DKO mouse testis. Scale bars, 25 μm. The red and black boxes are magnified images of the corresponding boxed areas of the lower magnification. Yellow arrowhead indicates ectopic localization of sperm near the basal lamina of *mDia1/3* DKO seminiferous tubule. (H) Quantification of the number of spermatozoa per seminiferous tubule in busulfan-treated testes of WT mice (20 tubules of three mice) or *mDia1/3* DKO mice (15 tubules of two mice) transplanted with *acro/act-EGFP* mice-derived testicular germ cell suspensions. Data are represented as mean ± SEM. ***P < 0.001 (Student *t* test). *acro/act-EGFP*, *acrosin/actin-*enhanced green fluorescent protein; DKO, double knockout; GFP, green fluorescent protein; HE, hematoxylin–eosin; mDia1, mammalian diaphanous homolog1; mDia3, mammalian diaphanous homolog3; n.s., not significant (*P* = 0.2675, Student *t* test); PAS, Periodic acid-Schiff; WT, wild-type.

To investigate how mDia1/3 double deficiency causes sperm abnormalities, we next performed histological analysis on spermatogenesis in the testes of adult WT and *mDia1/3* DKO mice. Paraffin sections of testis with Periodic acid-Schiff (PAS) staining revealed that, while numerous elongated spermatids aligned at the adluminal compartment of the seminiferous tubule in WT testes, the number of elongated spermatids in *mDia1/3* DKO testes is extremely low compared with WT testes (Fig 1A, red boxes). Notably, the heads of *mDia1/3* DKO sperm were not properly oriented toward the basal lamina of the seminiferous tubule (Fig 1A, black arrows in the lower red box). In addition, a fraction of *mDia1/3* DKO elongated spermatids exhibited ectopic localization in seminiferous tubules, as evidenced by their presence at the proximity of basal lamina (Fig 1A, black boxes). Moreover, we found a significant increase in apoptotic cell number in the *mDia1/3* DKO seminiferous tubule by TUNEL staining (S3A and S3B Fig). These results together suggest that mDia1/3 is indispensable for spermatogenesis.

We next attempted to figure out whether the mDia1/3 deficiency in germ cells or Sertoli cells is responsible for the above abnormal spermatogenesis phenotype observed in *mDia1/3* DKO mice. We first examined the expression of mDia1 and mDia3 in the seminiferous tubule by immunofluorescence staining. We found positive signals for mDia1 and mDia3 in vimentin-positive Sertoli cells [21] in the seminiferous tubule from adult WT mice (Fig 1B). These mDia1 and mDia3 signals were absent in testis sections from *mDia1* or *mDia3* knockout (KO) mice (S4A and S4B Fig), confirming the specificity of these signals. We also examined the expression of mDia3 in the WT seminiferous tubule during the spermatogenic cycle [22] and compared its localization with F-actin (S5A Fig). We found that mDia3 is expressed in Sertoli cells throughout the spermatogenic cycle, though its staining intensity and localization varies in different stages. For example, in stages IV–V, although the majority of mDia3 staining localizes in the cell body, a fraction of mDia3 staining with relatively low intensity (white arrows) colocalizes with F-actin bundles associated with apical ES. Similarly, in stages VI–VII, a fraction of mDia3 staining localizes close to F-actin bundles associated with basal ESs (white arrowheads), and the mDia3 staining becomes weak in stages X–XI. Specificity of these mDia3 signals was confirmed as they were mostly absent in testis sections from *mDia3* KO mice (S5B Fig).

We then examined the functional requirement of mDia1 and mDia3 expressed in Sertoli cells for spermatogenesis by germ cell transplantation (Fig 1C–1H), according to our previously established protocol [23]. We first transplanted testicular germ cell suspensions from WT or *mDia1/3* DKO mice to germ cell–free and Sertoli cell–only testes of *W/W*^*v*^ mice (Fig 1C), and examined spermatogenesis by hematoxylin–eosin (HE) staining of histological sections of the recipients (Fig 1D). We found that *W/W*^*v*^ mouse testes transplanted with *mDia1/3* DKO testicular cell suspensions had normal spermatogenesis, as did *W/W*^*v*^ mouse testes transplanted with WT testicular cell suspensions (Fig 1D). Quantification results revealed no significant difference of the spermatozoa number per seminiferous tubule in these transplanted mice (Fig 1E). Thus, mDia1/3 expression in germ cells is dispensable for spermatogenesis. As the reciprocal approach, we depleted germ cells of WT or *mDia1/3* DKO mice with busulfan treatment and transplanted testicular germ cell suspensions from *acrosin/actin-*enhanced green fluorescent protein (*acro/act-EGFP*) transgenic mice to each Sertoli cell–only testis (Fig 1F). We found that *acro/act-EGFP* testicular germ cells transplanted to busulfan-treated *mDia1/3* DKO testes mimicked abnormal sperm phenotypes seen in *mDia1/3* DKO testes, including a reduced number of elongated spermatid (Fig 1G, red box), ectopic localization near the basal lamina of the seminiferous tubule (Fig 1G, black box), and abnormal shape of the head (Fig 1G, black box, yellow arrowhead). Quantification revealed a significant decrease in the spermatozoa number per seminiferous tubule of busulfan-treated Sertoli-only *mDia1/3* DKO testes compared with WT testes (Fig 1H). Therefore, mDia1/3 function in Sertoli cells is required for normal spermatogenesis.

### Indispensable role of mDia1/3 in the architecture of cortical F-actin meshwork in the Sertoli cell

**Fig 2:**
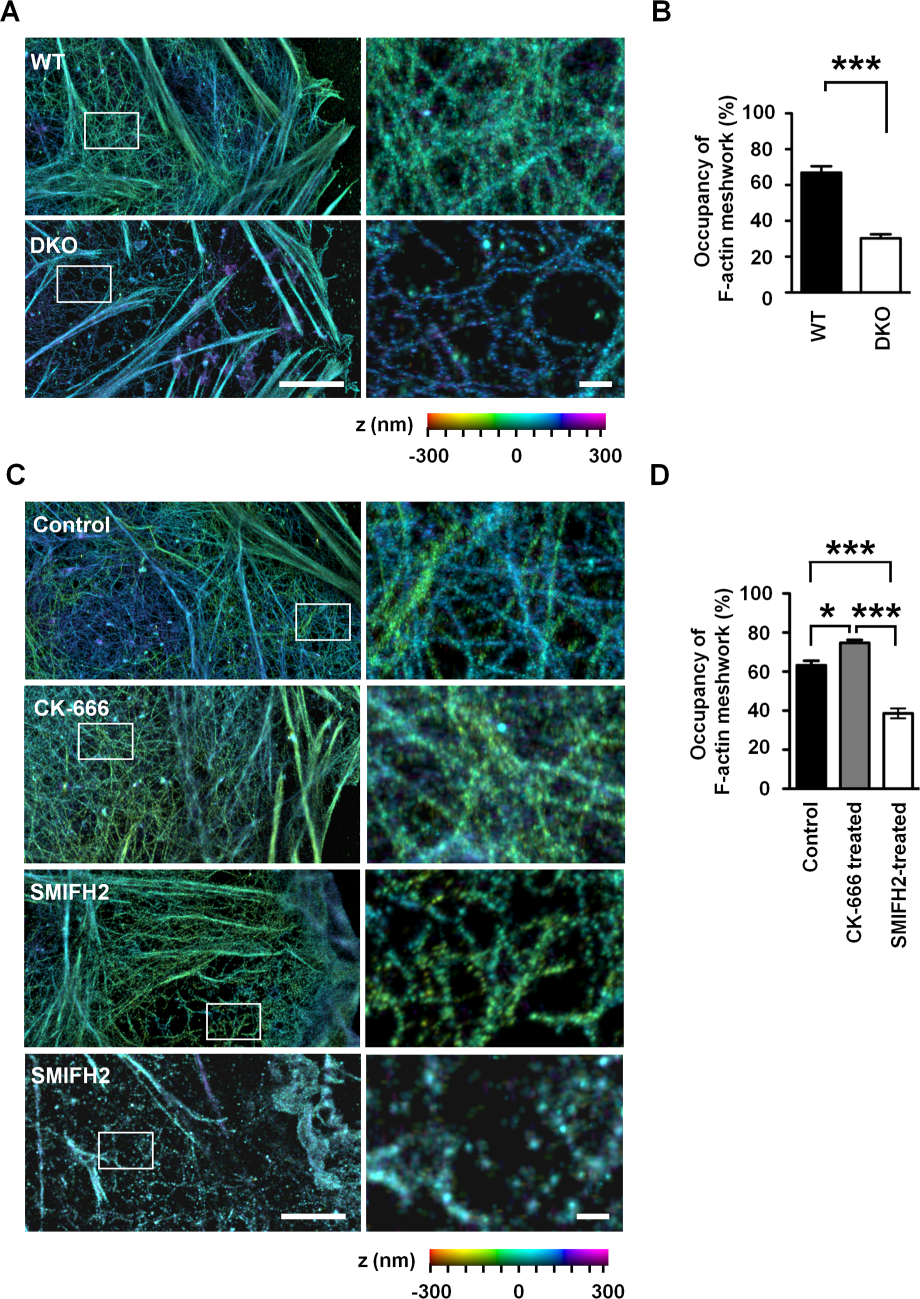
mDia1/3-dependent cortical F-actin meshwork in primary cultured Sertoli cells. (A) TIRF 3D-N-STORM imaging of the actin filaments in WT primary cultured Sertoli cells. The images on the right side are magnified images of the corresponding white boxed areas of the lower magnification. Color bar indicates the z dimension (−300 nm to 300 nm). Scale bars, 5 μm (left) and 500 nm (right). (B) Quantification of the ratio of F-actin meshwork occupancy per cell area in WT or *mDia1/3* DKO primary cultured Sertoli cells. Results of eight cells for each genotype from two independent experiments. Data are represented as mean ± SEM. ***P < 0.001 (Student *t* test). (C) Representative images of TIRF 3D-N-STORM imaging of the actin filaments in control WT primary cultured Sertoli cells or Sertoli cells treated with 30 μM SMIFH2 or 50 μM CK-666 for 1 h. Two SMIFH2-treated Sertoli cells with different extents of impaired cortical F-actin meshwork phenotype are shown. The images on the right side are magnified images of the corresponding white boxed areas of the lower magnification. Scale bars, 5 μm (left) and 500 nm (right). (D) Quantification of the ratio of F-actin meshwork occupancy per cell area in control, 50 μM CK-666–treated, or 30 μM SMIFH2-treated primary cultured Sertoli cells. Results of nine cells for control, eight cells for CK-666–treated, and 15 cells for SMIFH2-treated primary cultured Sertoli cells from two independent experiments. *P < 0.05, ***P < 0.001 (*P* = 0.017 for control versus CK-666–treated cells, *P* < 0.001 for control versus SMIFH2-treated cells, and *P* < 0.001 for CK-666–treated versus SMIFH2-treated cells; one-way ANOVA with post hoc test). DKO, double knockout; F-actin, filamentous actin; mDia1/3, mammalian diaphanous homolog1/3; SMIFH2, small molecule inhibitor of formin homology 2 domain; TIRF, total internal reflection; WT, wild-type; 3D-N-STORM, three dimensional-N-stochastic optical reconstruction microscopy.

Given the critical function of mDia1/3 expression in Sertoli cells, we then used confocal microscopy and examined F-actin structure in primary Sertoli cells cultured on gelatin-coated cover glass. We found that actin filaments in control WT Sertoli cells are composed of thick F-actin bundles and faintly stained actin filaments between these bundles (S6A Fig, left, and magenta line scanning). On the other hand, in *mDia1/3* DKO Sertoli cells, F-actin bundles were apparently retained, but the staining intensity of faintly stained actin filaments between these bundles was greatly reduced (S6A Fig, right, and green line scanning). To further resolve the architecture of faintly stained actin filaments in Sertoli cells at the nanoscale level, we next examined phalloidin-stained control WT cells with total internal reflection (TIRF) three dimensional-N-stochastic optical reconstruction microscopy (3D-N-STORM) superresolution microscopy [24]. We found that the faintly stained actin filaments between thick F-actin bundles observed in the cortices of WT cells by confocal microscope are actually a meshwork of actin filaments with a relatively large pore size of about 100 nm (Fig 2A, upper, white box). We then next examined the actin cytoskeleton organization of *mDia1/3* DKO Sertoli cells and found that the above cortical F-actin meshwork was markedly sparser, and there were areas devoid of actin filaments in these cells (Fig 2A, below, white box). Quantitative analysis of STORM images revealed that the occupancy of F-actin meshwork was significantly reduced in *mDia1/3* DKO Sertoli cells (Fig 2B). These results suggest that mDia1/3 contribute to the formation and maintenance of cortical F-actin meshwork in Sertoli cells.

We next sought to analyze the relative contribution between formins and Arp2/3 on cortical F-actin meshwork in Sertoli cells. To this end, we utilized pharmacological inhibitors for formins and Arp2/3. We found that treatment of WT cells with small molecule inhibitor of formin homology 2 domain (SMIFH2), a formin inhibitor [25], reduced cortical F-actin meshwork similarly as was observed in *mDia1/3* DKO cells (Fig 2C, third row). Notably, a fraction of SMIFH2-treated Sertoli cells showed total suppression of cortical F-actin meshwork with concomitant decrease in F-actin bundles (Fig 2C, fourth row). On the other hand, treatment of WT cells with CK-666, an Arp2/3 inhibitor [26], did not suppress the cortical F-actin meshwork (Fig 2C, second row). Quantitative analysis (Fig 2D) confirmed that treatment with SMIFH2 significantly suppressed cortical F-actin meshwork in Sertoli cells and additionally revealed that treatment of CK-666 resulted in a slight but significant increase of cortical F-actin meshwork. We speculate that the latter might be due to the increased availability of actin monomers for formins when Arp2/3-mediated actin polymerization is suppressed. These results together therefore suggest that the cortical F-actin meshwork in Sertoli cells is a structure dependent on the actin polymerization activity of formins, including mDia1 and mDia3, but not Arp2/3.

### mDia1/3 drives actin polymerization in cortical F-actin meshwork in Sertoli cells

**Fig 3:**
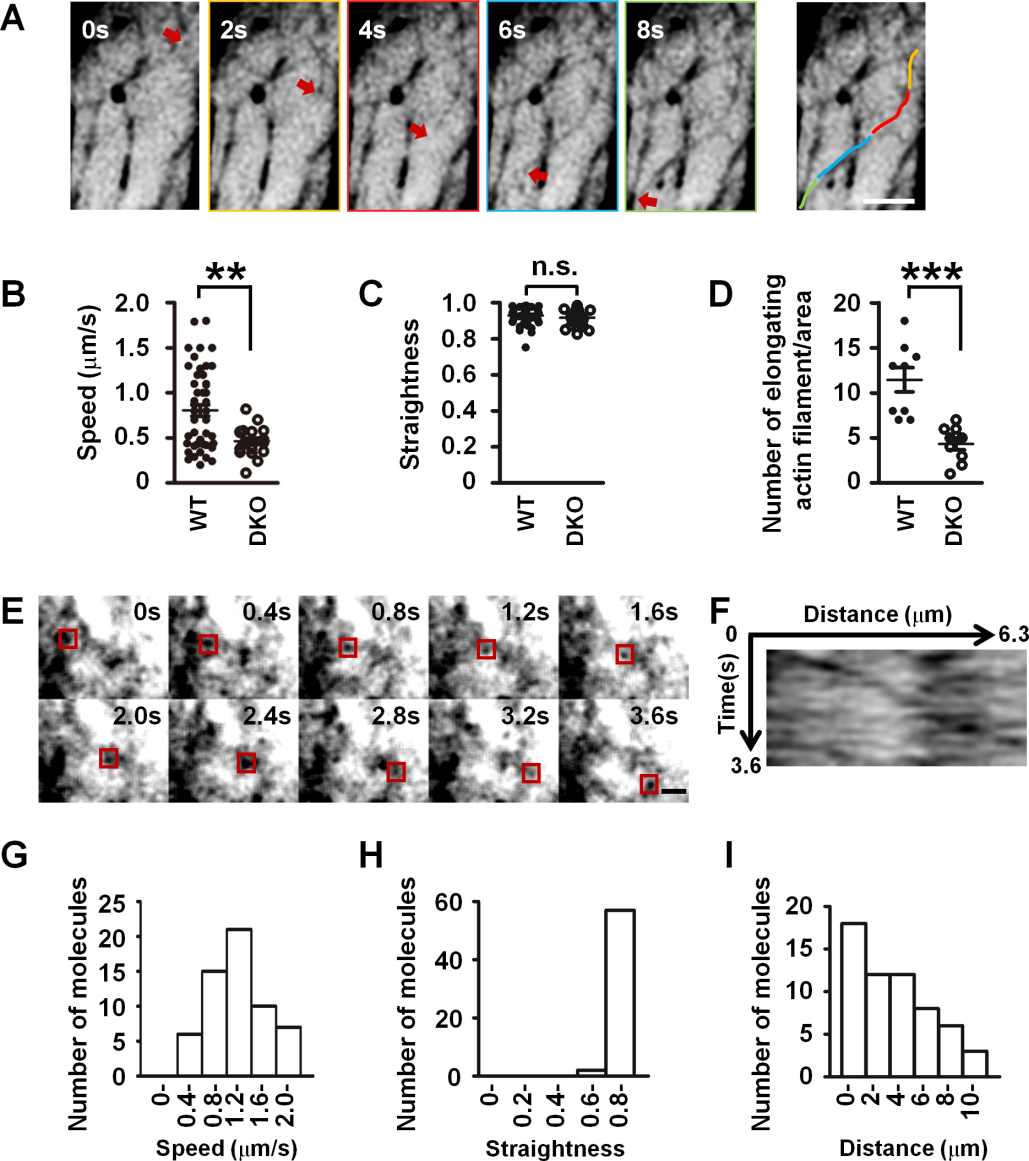
mDia1/3-dependent rapid dynamics of cortical F-actin meshwork in primary cultured Sertoli cells. (A) Time-lapse images of WT primary cultured Sertoli cell expressing LifeAct-EGFP. Red arrows indicate the tip of the elongating F-actin in the cortical meshwork. Time is shown in seconds. The colors in the right panel indicate the elongated actin filament at each time point in the left panel. Scale bar, 2 μm. (B) Quantification of the elongation speed of LifeAct-EGFP in WT and *mDia1/3* DKO Sertoli cells. Results from two independent experiments. Data are represented as mean ± SEM (*n* = 49 and 20 filaments from four WT and four *mDia1/3* DKO Sertoli cells, respectively). **P < 0.01 (*P* = 0.0013, Student *t* test). (C) Quantification of the straightness of the elongated F-actin in WT and *mDia1/3* DKO Sertoli cells. Results from two independent experiments. Data are represented as mean ± SEM (*n* = 49 and 20 filaments from four WT and four *mDia1/3* DKO Sertoli cells, respectively). (D) Quantification of the elongation frequency of cortical F-actin meshwork. Results from two independent experiments. Data are represented as mean ± SEM (*n* = 9 regions from three WT and three *mDia1/3* DKO Sertoli cells, respectively). ***P < 0.001 (*P* = 0.0002, Student *t* test). (E) Time-lapse images of WT primary Sertoli cell expressing EGFP-mDia3. Red boxes indicate a single molecule of EGFP-mDia3. Time is shown in seconds. Scale bar, 2 μm. (F) Kymograph of EGFP-mDia3 single molecule in Fig 3E. (G) Quantification of the processive moving speed of EGFP-mDia3 single molecules (*n* = 59 molecules from three cells). The result is representative of two independent experiments. (H) Quantification of the straightness of EGFP-mDia3 single molecules’ processive movement (*n* = 59 molecules from three cells). The result is representative of two independent experiments. (I) Quantification of the travel distance of EGFP-mDia3 single molecules (*n* = 59 molecules from three cells). The result is representative of two independent experiments. DKO, double knockout; EGFP, enhanced green fluorescent protein; F-actin, filamentous actin; mDia1/3, mammalian diaphanous homolog1/3; n.s., not significant (*P* = 0.3194, Student t test); WT, wild-type.

We next examined the dynamics of actin filaments in living primary cultured Sertoli cells. To this end, we introduced LifeAct-EGFP, a probe for F-actin [27], into primary cultured Sertoli cells. Utilizing spinning disk superresolution microscopy (SDSRM) [28], which allows fast image acquisition at the spatial xy-axis resolution of approximately 120 nm, we found that, while thick F-actin bundles are a relatively static structure, the cortical F-actin meshwork is an extremely dynamic structure, in which actin continually polymerizes and depolymerizes underneath the cortex of WT control cells (S1 Movie). Quantification of the dynamics of cortical meshwork actin filaments of WT Sertoli cells revealed that the F-actin elongated for a distance of several micrometers, and their extension rate was very fast, with a mean of 0.81 ± 0.06 μm/s (about 300 globular actin (G-actin) subunits incorporated per second) (Fig 3A and 3B, S2 Movie). The speed distribution further revealed at least two subpopulations of cortical meshwork actin filaments with different polymerization rates; the first subpopulation has a peak polymerization rate around 0.4 μm/s and the other subpopulation around 1.3 μm/s (Fig 3B). It should also be noted that F-actin elongation was highly straight (Fig 3C). On the other hand, in *mDia1/3* DKO cells, although the straightness of elongated F-actin was not affected (Fig 3C), the subpopulation of nascent actin filament with polymerization rate peak around 1.3 μm/s was absent (Fig 3B), and the number of elongation events was significantly reduced (Fig 3D). These results together suggested that cortical F-actin meshwork is a highly dynamic structure and its formation is dependent on mDia1 and mDia3.

Given the extremely rapid rate of actin polymerization observed in the cortical F-actin meshwork area and its impairment in *mDia1/3* DKO Sertoli cells, we hypothesized that actin filament polymerization in the meshwork is driven by mDia1 and mDia3. To test this hypothesis, we carried out single-molecule speckle imaging [29] of EGFP-mDia3 in living primary cultured WT Sertoli cells. The EGFP-mDia3 used in the experiment rescued the reduced cortical actin filament meshwork phenotype of *mDia1/3* DKO Sertoli cells (S7A–S7C Fig). Single-molecule speckle imaging with TIRF microscopy revealed fast and directional molecular movement of EGFP-mDia3 in the cortex of Sertoli cells (Fig 3E, S3 Movie and S4 Movie). Kymograph (Fig 3F) and quantification analyses of EGFP-mDia3 single-molecule speckles further indicated that the average speed of EGFP-mDia3 was extremely fast, with an average rate of 1.38 ± 0.06 μm/s (Fig 3G). In addition, the EGFP-mDia3 molecular movement was highly linear, similar to cortical meshwork actin filament (Fig 3H). Moreover, a single molecule of EGFP-mDia3 traveled for long distance—on average, 4.46 ± 0.40 μm (Fig 3I). Given that the spatial localization of a single molecule of EGFP-mDia3 in the cell cortex was similar to the cortical F-actin meshwork and the linear directional movement, long travel distance and fast movement speed of an EGFP-mDia3 single molecule were correlated with LifeAct-EGFP dynamics, and that the latter was diminished in the absence of mDia1 and mDia3, we concluded that cortical F-actin meshwork is an actin filament structure directly generated by mDia1/3 in primary cultured Sertoli cells. These data further substantiated that cortical F-actin meshwork is a mDia1/3-dependent structure.

### mDia1/3-dependent F-actin is indispensable for actomyosin bundles in Sertoli cells and formation of AJs between Sertoli cells and germ cells

**Fig 4:**
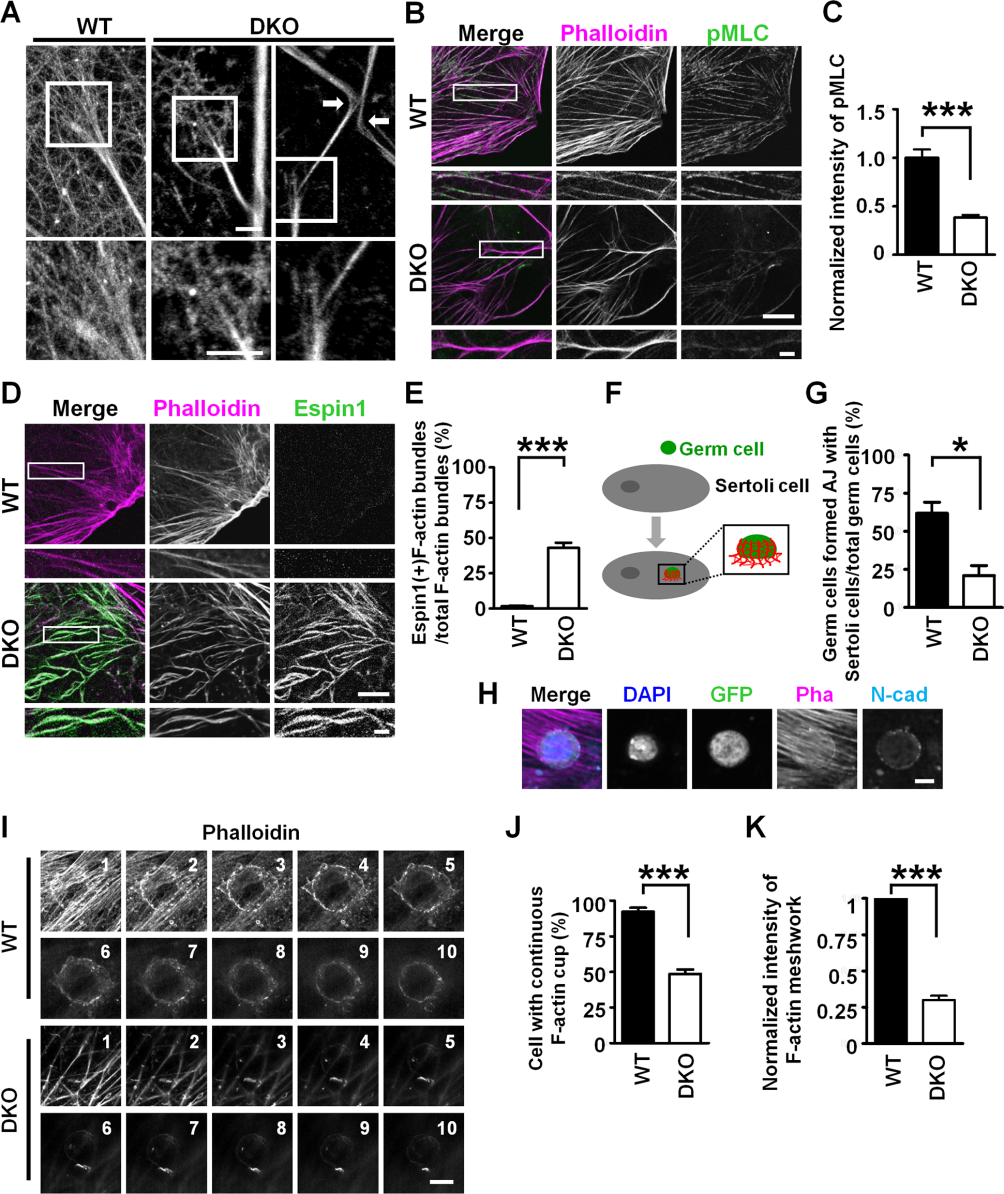
Loss of mDia1/3 in the Sertoli cell results in impaired actomyosin bundles and adherens junction. (A) TIRF N-STORM imaging of F-actin bundles of primary cultured Sertoli cells. Magnified images of the corresponding white box areas are shown below. Note that the F-actin bundle is a structure continuous with cortical actin filaments meshwork. Arrows indicate the bending F-actin bundles observed in *mDia1/3* DKO primary cultured Sertoli cells. Scale bars, 500 nm. (B) Primary cultured WT and *mDia1/3* DKO Sertoli cells stained with phalloidin (magenta) and pMLC (green). Note the reduced and discontinuity of pMLC staining on the F-actin bundles in the *mDia1/3* DKO Sertoli cell. Scale bars, 20 μm and 5 μm. White boxes are magnified and shown below the low magnification images. (C) Quantification of pMLC staining intensity. Staining intensity was normalized to the average intensity of control WT cells. Result is a sum of three independent experiments. Data are represented as mean ± SEM (*n* = 33 and 30 for WT cells and *mDia1/3* DKO cells, respectively). ***P < 0.001 (Student *t* test). (D) Primary cultured WT and *mDia1/3* DKO Sertoli cells stained with phalloidin (magenta) and espin1 (green). Note increased espin1 staining overlapped with F-actin staining in *mDia1/3* DKO Sertoli cell. Scale bars, 20 μm and 5 μm. White boxes are magnified and shown below the low magnification images. (E) Quantification of espin1-positive actin bundles per cell. Result is a sum of three independent experiments. Data are represented as mean ± SEM (*n* = 25 for WT Sertoli cells and *n* = 2 for *mDia1/3* DKO Sertoli cells). ***P < 0.001 (Student *t* test). (F) Schematic representation of Sertoli cell–germ cell coculture experiment. Green circle indicates germ cell isolated from 3-wk-old *EGFP* transgenic mouse testes, and gray ellipse indicates WT or *mDia1/3* DKO Sertoli cells. Red lines indicate actin filaments of the Sertoli cell, which surround the germ cell. (G) Percentage of germ cells formed adherens junction with Sertoli cells, as judged by N-cadherin staining on WT or *mDia1/3* DKO Sertoli cells in Sertoli cell–germ cell coculture experiments. Germ cells that had N-cadherin signals surrounding the germ cells were counted as adhesive cells. Result is the average of three independent experiments. Data are represented as mean ± SEM (*n* = 124 and 89 cells for WT and *mDia1/3* DKO, respectively). *P < 0.05 (*P* = 0.0306, Student *t* test). (H) Representative images of a germ cell judged as an adhesive cell with a WT Sertoli cell. (I) SDSRM images of Sertoli cell–germ cell in cocultured experiment stained with phalloidin. Images consist of 10 pairs of serial planes (z-step = 0.5 μm from bottom to top). Note that, while the germ cell attached to WT Sertoli cell is surrounded by actin filaments, such actin filaments are impaired in the germ cell attached to *mDia1/3* DKO Sertoli cell. (J) Quantification of the percentage of germ cells with actin filaments surrounding them in Sertoli cell–germ cell cocultured experiment. Result is from three independent experiments. Data are represented as mean ± SEM (*n* = 157 and 147 cells for WT and *mDia1/3* DKO, respectively). ***P < 0.001 (*P* = 0.0005, Student *t* test). (K) Quantification of the cortical F-actin intensity underneath the germ cell of the WT or *mDia1/3* DKO Sertoli cell in cocultured experiment. Staining intensity was normalized to the average intensity of control WT cells. Result is from two independent experiments. Data are represented as mean ± SEM (*n* = 23 and 17 cells for WT and *m1/3* DKO, respectively). ***P < 0.001 (*P* = 0.0001, Student *t* test). DKO, double knockout; *EGFP*, enhanced green fluorescent protein; F-actin, filamentous actin; mDia1/3, mammalian diaphanous homolog1/3; N-cad, neural cadherin; N-cadherin, neural cadherin; Pha, phalloidin; pMLC, phosphorylated myosin light chain; SDSRM, spinning disk superresolution microscopy; TIRF N-STORM, total internal reflection N-stochastic optical reconstruction microscopy; WT, wild-type.

In addition to the actin meshwork, thick actin bundles were seen in WT Sertoli cells and apparently similar actin bundles were observed also in *mDia1/3 DKO* Sertoli cells (Fig 2A). We therefore examined the origin and composition of these actin bundles. Higher magnification of TIRF-N-STORM images revealed that thick F-actin bundles of Sertoli cells are structurally continuous with the cortical F-actin meshwork both in WT and *mDia1/3* DKO Sertoli cells (Fig 4A, left and middle). However, we noted that whereas these thick F-actin bundles were mostly intact in WT cells (Fig 4A, left), those in *mDia1/3* DKO cells were occasionally bent (Fig 4A, right). Utilizing confocal microscopy, we found that most of F-actin bundles in primary cultured WT Sertoli cells were co-stained for phosphorylated myosin light chain (pMLC) (Fig 4B, top) and thus represent contractile actomyosin bundles [30]. On the other hand, the pMLC staining intensity in F-actin bundles was greatly reduced in *mDia1/3* DKO Sertoli cells (Fig 4B, bottom). Quantification of pMLC staining showed a significant decrease in *mDia1/3* DKO cells (Fig 4C). To identify actin bundles observed in *mDia1/3* DKO seminiferous tubules, we next performed immunostaining for espin1, an actin bundling protein associated with noncontractile F-actin bundles [31]. We found that most of F-actin bundles in primary cultured Sertoli cells from *mDia1/3* DKO mice were associated with espin1 (Fig 4D). Quantification further revealed that F-actin bundles associated with espin1 comprised about 50% of total F-actin bundles in *mDia1/3* DKO Sertoli cells but were rarely observed in WT Sertoli cells (Fig 4E). These results together indicate that mDia1/3 are indispensable not only for the formation and maintenance of the cortical F-actin meshwork but also for the generation of contractile actomyosin bundles in Sertoli cells.

To examine how our findings on F-actin structures in Sertoli cells cultured in vitro may reflect those in vivo, we next investigated the F-actin structures in Sertoli cells in intact seminiferous tubules. To this end, we selectively fluorescently labeled Sertoli cells in vivo by microinjection of lentivirus-expressing LifeAct-EGFP to seminiferous tubules, according to our method reported previously [32]. We then dissected and untangled the lentivirus-injected seminiferous tubules, fixed and prepared them for imaging ex vivo [33]. Deconvoluted Z-stack images with spinning disk confocal microscopy showed that, although the morphology of intact Sertoli cells was different from that of cultured Sertoli cells, their F-actin structures imaged with LifeAct-EGFP consisted of at least two different F-actin structures, the thick F-actin bundles and thin F-actin meshwork (S8 Fig, S5 Movie). These results suggest that the overall F-actin architectures of Sertoli cells in intact seminiferous tubules are largely similar to those found in cultured Sertoli cells.

Recent studies suggested an important role of contractile actomyosin bundles in the regulation of cell–cell AJ [34,35]. Given that actomyosin bundles were impaired in primary cultured *mDia1/3* DKO Sertoli cells, we next utilized an in vitro reconstitution experiment and investigated the impact of the loss of mDia1/3 on the formation of the AJ between a Sertoli cell and a round spermatid cell (Fig 4F). In this system, germ cells isolated from the approximately 3-wk-old *EGFP* transgenic mice testes containing mostly round spermatids were added onto the primary cultured Sertoli cells and further cocultured for 24 h to allow the formation of Sertoli cell–round spermatid AJ. Immunocytochemistry of an AJ protein, N-cadherin, revealed that while approximately 50% of the round spermatids on WT primary Sertoli cells formed N-cadherin positive cell–cell adhesion, less than 20% of germ cells formed N-cadherin positive cell–cell adhesion with *mDia1/3* DKO primary Sertoli cells (Fig 4G and 4H). In addition, we found that the continuity of F-actin on the round spermatid–Sertoli cell interface was often disrupted in the *mDia1/3* DKO Sertoli cell (Fig 4I and 4J). Quantitative analysis further revealed that the density of the cortical F-actin meshwork beneath the germ cell in WT Sertoli cells was compromised in *mDia1/3* DKO Sertoli cells (Fig 4I and 4K). These results together suggest that mDia1/3-mediated F-actin is critical for formation of the AJ between Sertoli cells and round spermatids.

### mDia1/3 regulate the Sertoli cell–germ cell AJ and ES junction in the seminiferous tubule through the control of F-actin bundling in the Sertoli cell

**Fig 5:**
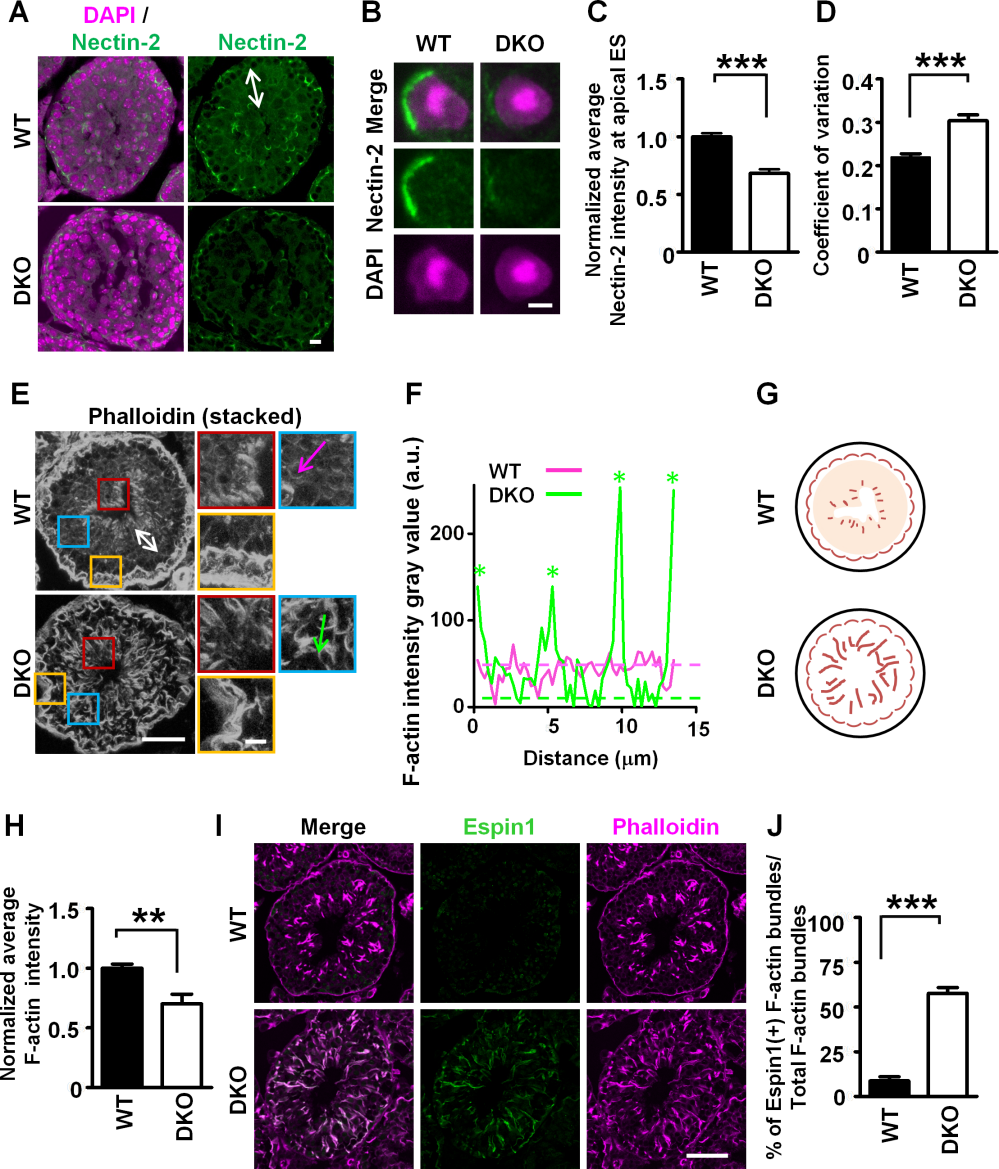
Loss of mDia1/3 results in impaired Sertoli cell–germ cell adhesion and aberrant and ectopic actin bundling in seminiferous tubule. (A) Immunofluorescence staining for nectin-2 (green) and nuclear counterstaining with DAPI (magenta). White two-way arrow indicates the area where round spermatids are located in seminiferous tubule. (B) High magnification of concentrated nectin-2 staining at apical ES junction between Sertoli cell and elongated spermatids. Note that while signals for apical ES junction nectin-2 concentrated around the head of the elongated spermatids were relatively continuous in WT seminiferous tubule, nectin-2 signal around the head of elongated spermatids is often lower than that of the WT and its continuity around the spermatids head is impaired in *mDia1/3* DKO apical ES junction. Scale bars, 100 μm (A) and 2.5 μm (B). (C) Quantification of the normalized nectin-2 staining intensity at the apical ES junction. Result is a sum of three independent experiments. Data are represented as mean ± SEM (*n* = 35 and 39 cells for WT and *mDia 1/3* DKO, respectively). ***P < 0.001 (Student *t* test). (D) Quantification of the coefficient of variation of nectin-2 staining intensity along the apical ES junction. Result is from three independent experiments. Data are represented as mean ± SEM (*n* = 35 and 39 cells for WT and *mDia 1/3* DKO, respectively). ***P < 0.001 (Student *t* test). (E) WT (upper) and *mDia1/3* DKO (lower) adult seminiferous tubule stained with phalloidin. The white two-way arrow indicates the round and elongated spermatids zone of the WT seminiferous tubule, with F-actin faintly stained. Red, yellow, and blue boxes are magnified images of the corresponding boxed areas of the lower magnification. Scale bars, 100 μm (left) and 10 μm (right). The lines (magenta and green arrows) in the blue boxes were used to calculate the fluorescence intensity. (F) Line scans of the fluorescence intensity for phalloidin staining. Fluorescence-intensity profiles along the magenta and green arrows shown in E are shown. Green asterisks indicate ectopic thick F-actin bundles observed in *mDia1/3* DKO seminiferous tubule. (G) Simplified scheme summarized results of the observation in Fig 5E–5F. (H) Quantification of total F-actin staining intensity. Result is a sum of three independent experiments. Data are represented as mean ± SEM (*n* = 21 and 18 for WT and *mDia 1/3* DKO, respectively). **P < 0.01 (*P* = 0.001, Student *t* test). (I) WT (upper) and *mDia1/3* DKO (lower) adult testis immunostained for espin1 (green) and F-actin (magenta). Note that the espin1 staining in the *mDia1/3* DKO seminiferous tubule was abnormally strong and colocalized to phalloidin staining of aberrant and ectopic thick F-actin bundles observed in the *mDia1/3* DKO seminiferous tubule. Scale bar, 100 μm. (J) Quantification of espin1-positive actin bundles. Result is a sum of three independent experiments. Data are represented as mean ± SEM (*n* = 12 seminiferous tubules for WT mice and *n* = 17 seminiferous tubules for *mDia1/3* DKO mice). ***P < 0.001 (Student *t* test). a.u., arbitrary unit; DKO, double knockout; ES, ectoplasmic specialization; F-actin, filamentous actin; mDia1/3, mammalian diaphanous homolog1/3; WT, wild-type.

Because the above findings showed the role of mDia1/3 in the formation of AJ between Sertoli cells and germ cells in vitro, we next examined if the mDia1/3 deficiency in Sertoli cells affects these cell–cell interactions in the seminiferous tubule in vivo. To this end, we performed immunostaining for nectin-2, an adhesion molecule exclusively expressed in Sertoli cells [36,37]. Composite Z-stack images of nectin-2 in WT seminiferous tubules showed nectin-2 signals at the AJ between a Sertoli cell and a round spermatid (see the area shown by the white two-way arrow in Fig 5A, WT) and concentration of nectin-2 at the apical ES junction between Sertoli cells and elongated spermatids and basal ES between Sertoli–Sertoli cells (Fig 5A). On the contrary, nectin-2 signal at the boundary between Sertoli cells and round spermatids was significantly reduced in *mDia1/3 DKO* seminiferous tubules (Fig 5A, below). We also stained for nectin-2 in WT and *mDia1/3* DKO seminiferous tubules throughout the spermatogenic cycles and observed consistent results (S9 Fig). Moreover, high magnification images (Fig 5B) revealed that the accumulation (Fig 5C) and the continuity (Fig 5D) of concentrated nectin-2 signals at the apical ES junction were severely impaired in the *mDia1/3* DKO seminiferous tubule. These results indicate that mDia1/3 are indispensable for both AJ and apical ES junction in the seminiferous tubule.

It is known that while conventional AJs are associated with contractile actomyosin bundles [34,35], ES junctions are typified by the presence of highly dense noncontractile F-actin bundles in the Sertoli cell [13,14]. To further resolve the F-actin architecture of WT and *mDia1/3* DKO seminiferous tubules, we next conducted phalloidin staining and examined F-actin structures by confocal imaging (Fig 5E). Composite Z-stack images of F-actin in WT seminiferous tubules show that, while thick F-actin bundles specifically localize to the apical ES junction along the elongated spermatid head (Fig 5E, above, red box) and basal ES at this stage (Fig 5E, above, yellow box), thick F-actin bundles were intriguingly increased in number and intensity and aberrantly distributed over the whole region of the *mDia1/3* DKO seminiferous tubule (Fig 5E, below). Moreover, whereas the background F-actin staining in the area where round spermatids are located as shown by the white two-way arrow, is relatively high in WT seminiferous tubules (Fig 5E, above), they were strongly reduced between the ectopic thick F-actin bundles in *mDia1/3* DKO seminiferous tubules (Fig 5E, blue box). Line scanning of the F-actin intensity in the round spermatid area (Fig 5E, blue box, magenta and green lines) clearly showed the stark difference between the faintly stained F-actin density in WT and *mDia1/3* DKO Sertoli cells (Fig 5F). Based on these observations, we summarized our results in a simplified diagram (Fig 5G). Quantification revealed that the total F-actin intensity per seminiferous tubule was significantly reduced in *mDia1/3* DKO seminiferous tubules compared with that of WT seminiferous tubules (Fig 5H).

To further clarify the identity of abnormal thick actin bundles specifically observed in *mDia1/3* DKO seminiferous tubules, we next performed immunostaining of espin1. We found that whereas espin1 staining is relatively weak and confined to the area around the head of elongated sperm in control WT seminiferous tubules at stages X–V of the spermatogenic cycle, it showed persistent and abnormally strong signals in *mDia1/3* DKO seminiferous tubules, which are mostly colocalized with ectopic F-actin bundles (Fig 5I and S10 Fig). Quantification showed that F-actin bundles associated with espin1 comprised about 60% of total F-actin bundles in *mDia1/3* DKO seminiferous tubules but were rarely observed in WT seminiferous tubules (Fig 5J). These findings together suggested that the loss of mDia1/3 impaired normal F-actin structures in both AJs and apical ES junctions and induced the formation of ectopic aberrant espin1-containing F-actin bundles in seminiferous tubules.

## Discussion

Despite advances in our understanding of formins’ biochemical activity, how and where formins generate F-actin and what forms of F-actin structures they make in a variety of cell types and tissues remain largely unknown. Technically, the presence of abundant F-actin bundles in adherent cells makes the observation of the fine cortical actin network challenging. In this work, we found that although *mDia1* KO male mice and *mDia3* KO male mice were fully fertile, *mDia1/3* DKO male mice were infertile, suggesting the functional redundancy between the two mDia isoforms. Starting with the Sertoli cell–intrinsic defects in spermatogenesis in *mDia1/3* DKO mice, we examined how the formins mDia 1 and mDia3 contribute to actin cytoskeleton structure and dynamics in Sertoli cells and spermatogenesis. We employed superresolution microscopy, 3D-N-STORM, to unravel the nanoscale F-actin architecture in Sertoli cells and found that the actin cytoskeleton in these cells is strongly dependent on formins. We observed at least two types of actin cytoskeleton structure coexisting in primary cultured WT Sertoli cells, the thin cortical F-actin meshwork and the thick actomyosin bundles. Notably, two types of actin filaments were also seen in Sertoli cells in situ in testis tubules. High magnification superresolution images further revealed that these two types of F-actin are of continuous structure. In contrast to the previous findings on cortical F-actin meshwork in cells cultured in suspension [18, 19], we found that the cortical meshwork of Sertoli cells on the substrate has a larger pore size and is largely dependent on formin activity, including mDia1/3, but not on Arp2/3. Utilizing spinning disk superresolution live imaging [28], we further found that the cortical F-actin meshwork is a highly dynamic structure. Quantitative analysis of the cortical F-actin network in cultured Sertoli cells revealed two subpopulations in the cortical F-actin meshwork, the first subpopulation with the polymerization rate peak at about 0.4 μm/s and the second subpopulation with polymerization rate peak at about 1.3 μm/s. In *mDia1/3* DKO Sertoli cells, the latter subpopulation was totally absent. Therefore, the subpopulation of newly polymerized actin with faster speed is dependent on mDia1/3 activity. Moreover, we found that the dynamic of a single mDia3 molecule in living Sertoli cells on a substrate is similar to that of the second subpopulation of cortical F-actin meshwork, with polymerization rate of about 1.3 μm/s. Given this correlation, it is likely that mDia1 and mDia3 generate a particular fraction of cortical F-actin meshwork with fast dynamics in Sertoli cells. We speculate that the other fraction of cortical F-actin meshwork is mediated by other formins expressed in Sertoli cells, such as formin1 [38], because cortical F-actin meshwork was strongly suppressed by treatment with the formin inhibitor, SMIFH2 [25].

Intriguingly, we also found that actomyosin bundles are impaired in *mDia1/3* DKO Sertoli cells. In the absence of mDia1 and mDia3, noncontractile actin bundles containing espin1 replaced contractile actomyosin bundles in Sertoli cells. The mechanism by which mDia1/3-generated actin filaments are preferentially incorporated to actomyosin bundles and not to espin1-containing actin bundles remains unclear. We previously demonstrated that mDia1 helically rotates at the barbed end of the actin filament [39] and proposed that it might cause the twist of F-actin [40]. Because it was reported that the twisting of an actin filament affects its conformation and influences subsequent binding of actin binding proteins (ABPs), including myosin II [41], we speculate that mDia1 might affect the conformation of an actin filament that allows efficient binding of myosin II. It should also be noted that filament spacing in actin bundles is an architectural feature dependent on the actin bundling proteins. It was reported that the incorporation of small-sized (about 8 nm) espin1 molecules into F-actin results in compact F-actin bundling, which is segregated from F-actin containing contractile myosin of the larger size [42]. We speculate that long and straight actin filaments generated by mDia1/3 in the cortical meshwork may not be favorable for espin1 binding and instead may allow larger protein cross-linking to the formation of actomyosin bundles. A previous study reported that espin-associated F-actin bundles assemble into highly ordered, densely packed bundles [13]. On the other hand, the actomyosin bundle is a structure of parallel filaments of opposite polarity spaced apart by myosin [43,44]. Therefore, it is possible that espin-based actin bundles contain a larger number of actin filaments per bundle than actomyosin in *mDia1/3* DKO Sertoli cells. Nevertheless, they are apparently more fragile than actomyosin bundles as they are occasionally bent, as observed in *mDia1/3* DKO Sertoli cells in this study.

Actomyosin is a contractile structure generating force in various cellular contexts, and its importance in maintaining the cell–cell AJ has been recently reported [34,35]. Consistently, we found that formation of the AJ was impaired in the *mDia1/3* DKO seminiferous tubule, as evidenced by suppressed cell adhesion between round spermatids and Sertoli cells in vitro and reduced nectin-2 staining in Sertoli cell–germ cell junction in vivo. It is plausible that mDia1/3 supply cortical actomyosin bundles to encircle spermatids that may limit the diffusion of AJ proteins and promote their clustering in Sertoli cells, as reported as a prerequisite event for cell–cell junction formation in other systems [17,45]. Notably, a small fraction of germ cells that could form cell–cell junctions with *mDia1/3* DKO Sertoli cells in vitro showed impaired continuity of F-actin in the proximity of cell–cell junctions. Therefore, mDia1/3-mediated actomyosin bundles in Sertoli cells also play a role in stabilization and maintenance of cell–cell junctions. It should also be noted that in addition to AJs, apical ES junctions were also severely impaired in *mDia1/3* DKO mice. As the ES junction is formed subsequent to the AJ during sperm development, we speculate that proper AJ is a prerequisite for ES junction formation. One intriguing question is how the actomyosin bundles generated by mDia1/3 localize specifically to the AJ and exert their function there. Several models were previously proposed for the generation of actomyosin bundles at the AJ [34,46]. Because we observed mDia1/3-dependent actin polymerization throughout the Sertoli cell cortex, our results support a model that mDia1/3-depedent actomyosin bundles are generated in the cortex but the localization to the AJ is mediated by other side-binding ABPs, such as α-catenin [47].

Sertoli cells make adhesion with developing germ cells and contribute to spermatogenesis by providing structural and functional support [8]. Thus, proper adhesions between Sertoli cells and germ cells are indispensable for normal spermatogenesis. Involvement of F-actin in sperm development was also previously reported [48]. However, the detailed molecular mechanism of F-actin action in spermatogenesis, especially in the supporting Sertoli cells, was largely unknown. In this work, we demonstrated that mDia1/3-dependent cortical F-actin meshwork and contractile actomyosin in Sertoli cells are critical for spermatogenesis through the regulation of Sertoli cell–germ cell adhesion.

In summary, here we have combined genetic and pharmacological manipulation of formins with superresolution and single-molecule imaging, and revealed a previously unsuspected requirement for the two formins, mDia1 and mDia3, in sperm morphogenesis and male fertility. Importantly, mDia1/3 catalyze the formation of cortical F-actin meshwork and actomyosin bundles indispensable for the formation and function of AJs and apical ES junctions essential for proper spermatogenesis (S11 Fig). The function of mDia unraveled here thus sheds a light on the importance of the F-actin structure and dynamics in Sertoli cells for spermatogenesis and paves a way for molecular dissection of its regulatory mechanisms.

## Materials and methods

### Materials

Primary antibodies used were rabbit anti-mDia1 polyclonal (LifeSpan BioSciences, Seattle, WA), rabbit anti-mDia3 polyclonal (SIGMA-Aldrich, St. Louis, MO), chicken anti-vimentin polyclonal (Millipore, Burlington, MA), rabbit anti-GFP polyclonal (MBL, Nagoya, Japan), goat anti-nectin-2 polyclonal (Santa Cruz Biotechnology, Dallas, TX), rabbit anti-espin1 polyclonal (#PB538, a gift from Dr. Bechara Kachar, NIH) [49], mouse anti-espin1 monoclonal, mouse anti-N-cadherin monoclonal (BD Bioscience, San Jose, CA), and mouse anti-phospho-myosin light chain2 (Ser19) monoclonal (Cell Signaling, Danvers, MA). Inhibitors used were SMIFH2 (Tocris, Bristol, United Kingdom) and CK-666 (Tocris, Bristol, UK).

### Animals

*mDia1* KO mice were generated and backcrossed to C57BL/6N mice for more than 10 generations, as previously described [3]. *mDia3* KO mice and *mDia1/mDia3* DKO were generated and backcrossed to *C57BL/6N* mice for more than 10 generations, as previously described [5]. *C57BL/6-Tg* (*CAG-EGFP*) mice were purchased from the local supplier (Japanese SLC, Hamamatsu, Japan) and *acro/act-EGFP* [*TgN(acro/act-EGFP)OsbC3-N01-FJ002*] mice were kindly provided by Dr. Masaru Okabe (Osaka University). All animal care and use were in accordance with the United States National Institutes of Health *Guide for the Care and Use of Laboratory Animals* and were approved by the Institutional Animal Care and Use Committee of Kyoto University Graduate School of Medicine.

### Primary culture of Sertoli cells

Testes from 12-wk-old WT or *mDia1/3* DKO mice were collected in cold Hanks’ balanced salt solution (HBSS; Invitrogen, Carlsbad, CA) and placed on ice. After removal of the tunica albuginea, seminiferous tubules were dissociated from the testis and transferred immediately into 1 mL of 1 mg/mL collagenase in HBSS. Tubules were incubated for 15 min at 37°C in a water bath, then washed with HBSS three times. Collagenase-treated tubules were then dissociated in a mixture of 0.8 mL of 0.25% trypsin and 0.2 mL of 7 mg/mL DNase in HBSS for 10 min at 37°C in a water bath. A total of 5 mL of 10% FBS-contained Iscove’s modified Dulbecco’s medium (IMDM; Invitrogen, Carlsbad, CA) were subsequently added to the trypsinized cells and mixed by gently pipetting. The mixture was then centrifuged at 400*g* for 5 min. Cells were resuspended in 10% FBS-contained IMDM, counted, and then plated at a concentration of 8 × 10^5^ cells/mL per well on a 0.2% gelatin-coated cover glass. Cells were allowed to settle for 24 h in 5% CO_2_ at 37°C. Nonattached cells were washed out with serum-free IMDM four times. Finally, 1 mL/well of IMDM containing 10% FBS was added the culture and cells were cultured for an additional 48 h in a CO_2_ incubator, as above.

### In vitro fertilization

In vitro fertilization was performed as described previously [50]. In brief, female *B6D2F1* mice were superovulated by intraperitoneal injections of equine chorionic gonadotropin (eCG; Teikoku Zoki, Tokyo, Japan) and human chorionic gonadotropin (hCG; Teikoku Zoki, Tokyo, Japan) at 48-h intervals. Ovulated eggs were recovered 13 h after the hCG injection and placed in a 200-μL drop of modified Krebs–Ringer bicarbonate solution (TYH medium) [51] containing glucose, sodium pyruvate, bovine serum albumin, and antibiotics (0.05 mg/mL penicillin, 100 IU/mL streptomycin). Fresh spermatozoa from the cauda epididymis were dispersed in a 200-μL drop of TYH medium, diluted to 1×10^6^ spermatozoa/mL, and incubated for 1.5 h to induce capacitation. An aliquot of capacitated spermatozoa from control WT mice and *mDia1/3* DKO mice were then added to the eggs at 2×10^5^ spermatozoa/ml for IVF. The mixture was incubated for 8 h at 37°C under 5% CO_2_ in air. Fertilization was confirmed by pronuclear formation. For observation of sperm–zona binding, egg masses were treated with bovine testicular hyaluronidase (175 U/mL; Sigma-Aldrich, St. Louis, MO) for 5 min to remove the cumulus cells. The cumulus-free eggs were placed in a 200-μL droplet of TYH medium and inseminated as described. After 30 min of incubation, sperm binding to the zona pellucida of the eggs was observed under an IX-70 microscope (Olympus, Tokyo, Japan).

### Sperm count

The cauda epididymis was collected and minced with a razor blade in 1 mL PBS. The suspension was filtered through 70-μm-nylon mesh (Corning, Corning, NY). The number of spermatozoa was counted using a hemocytometer.

### Sperm motility analysis

Cauda epididymal spermatozoa were suspended and incubated in TYH medium. Sperm motility was then measured using the CEROS sperm analysis system (software version 12.3; Hamilton Thorne Biosciences, Beverly, MA). Analysis settings were as described previously [52]. The percentage of hyperactivated spermatozoa was analyzed as described previously [53].

### Separation and fixation of spermatozoa from mouse testis

Mature sperm were collected from cauda epididymis by pricking with a fine needle and rinsed twice with PBS. Sperms were then plated on slide glasses, air-dried, and fixed with 4% PFA/PBS for 15 min at room temperature. Sperm morphology was observed on a laser scanning confocal imaging system (Leica SP5) with an oil immersion objective lens (100×1.4 numerical aperture).

### Transplantation

To enrich spermatogonial stem cells in donor testicular cell suspension, experimental cryptorchidism was surgically induced in donor mice [54]. Three to six weeks after the surgery, testis cells were dissociated into single-cell suspensions by two-step enzymatic digestion using collagenase type IV and trypsin (SIGMA-Aldrich, St. Louis, MO), as described previously [23]. The dissociated WT or *mDia1/3* DKO cells were then transplanted into *W/W*^*v*^ recipient mice (Japan SLC, Hamamatsu, Japan) through the efferent duct. For reciprocal transplantation, testis cells from cryptorchid *acro/act-EGFP* transgenic mice were transplanted into busulfan-treated control WT or *mDia1/3* DKO mice through the efferent duct. Approximately 5 or 10 μL was introduced into the testes, respectively. Each injection filled 75%–85% of the seminiferous tubules.

### Histology and immunohistochemistry

Testes or epididymides were fixed with 4% PFA at 4°C overnight. For paraffin sections, the testes or epididymides were embedded in paraffin and cut into sections at 4-μm thickness. For frozen sections, the testes were cryoprotected in 0.1 M PB containing 30% sucrose, frozen in Tissue-Tek OCT compound (Sakura-finetek, Tokyo, Japan) in dry ice, and then were cut into sections at 12-μm thickness using cryostat. HE and PAS staining were performed according to standard protocol. For immunohistochemistry, cryosections were washed with PBS three times for 5 min each. Antigen retrieval for mDia1, nectin-2, espin1, and vimentin staining was carried out by boiling the sections in 10 mM citrate buffer, pH 6.0, with a pressure cooker. Sections were incubated with the blocking buffer (PBS containing 1% normal donkey serum (Jackson laboratory, Bar Harbor, ME) or 1% normal goat serum (Jackson laboratory, Bar Harbor, ME) and 0.3% TritronX-100) for 1 h at room temperature. Sections were then incubated with the primary antibody diluted in blocking buffer. After three washes with 0.3% Triton X-100 in PBS, sections were incubated with appropriate secondary antibodies conjugated to Alexa Fluor 488, Alexa Fluor 555, Alexa Fluor 594, or Alexa Fluor 633 (Invitrogen, Carlsbad, CA). Hoechst 33342 (Invitrogen, Carlsbad, CA) or DAPI (Molecular Probes, Eugene, OR) was used for nuclear staining. Phalloidin conjugated with Alexa 488, 546, 555, or 647 (Invitrogen, Carlsbad, CA) was used for the staining of F-actin. Peanut agglutinin (PNA) conjugated with TRITC (SIGMA-Aldrich, St. Louis, MO) was used for the staining of round and early elongated spermatids acrosome. TUNEL staining was performed according to the manufacturer’s instructions using ApopTag Fluorescein Direct In Situ Apoptosis Detection Kit (Millipore, Burlington, MA). Fluorescent images were acquired with a laser scanning confocal imaging system (Leica SP5) equipped with 100× NA 1.4 HCX PL APO CS oil immersion objective lens (Leica, Wetzlar, Germany). Images were processed using Adobe Photoshop CS5 software (Adobe, San Jose, CA).

### Quantification analysis of nectin-2 intensity, F-actin intensity, and proportion of espin1-positive F-actin bundles

For quantification of nectin-2 intensity, nectin-2 staining signals associated with the apical ectoplasmic specialization junction were selectively visualized by setting a threshold, and then the average intensity per pixel was analyzed by ImageJ software (http://rsbweb.nih.gov/ij). For coefficient of variation of nectin-2, a line scan of the nectin-2 staining signal along the apical ectoplasmic junction was performed using ImageJ software by drawing a line, and the intensity values along the line were obtained using the Plot Profile. Coefficient of variation of nectin-2 staining intensity along the apical ectoplasmic junction were then calculated as the ratio of the standard deviation and average staining intensity. Line scan for measurement of F-actin intensity was performed using ImageJ software by drawing a line on the seminiferous tubule, and the intensity values along the line were obtained using the Plot Profile. Plots were generated based on the data obtained by ImageJ using Prism software (GraphPad Software, San Diego, CA). For quantification of seminiferous tubule F-actin intensity, region of interest (ROI) was defined to specifically include the seminiferous tubule but not the myoid cell layer, and average F-actin intensity per pixel was then analyzed by ImageJ software. Averages of F-actin intensity per pixel of WT and *mDia1/3* DKO seminiferous tubules were then generated based on the data obtained by ImageJ using Prism software (GraphPad Software, San Diego, CA). For quantification of the proportion of espin1-positive actin bundles, a binary image of F-actin bundles and espin1 were generated by setting a threshold. The two images were then merged and the pixel number of total F-actin bundles and espin1 signals that are colocalized with F-actin bundles was analyzed. The proportion of espin1-positive F-actin bundles were then calculated as a ratio of the pixel number of espin1-associated F-actin bundles and total F-actin bundles for seminiferous tubules. Averages of the proportion of espin1-positive actin bundles of WT and *mDia1/3* DKO seminiferous tubules were then generated using Prism software (GraphPad Software, San Diego, CA).

### Fixation and phalloidin staining for TIRF 3D-N-STORM superresolution imaging

Sertoli cells were cultured for 72 h on 25 mm round, No. 1.5 fiducialated cover glass (Hestzig, Leesburg, VA; #600-100AuF) for TIRF 3D-N-STORM superresolution imaging. For the fixation, the cells were briefly washed with 2.5 mL of prewarmed (37°C) PBS and fixed and permeabilized with 0.3% glutaraldehyde (EM Grade; Electron Microscopy Sciences, Hatfield, PA) and 0.25% Triton X-100 in 1 mL of cytoskeleton buffer (10 mM MES, pH 6.1, 150 mM NaCl, 5 mM EGTA, 5 mM glucose, and 5 mM MgCl_2_) for 1.5 min. The second fixation step was performed with 2% glutaraldehyde in 1 mL of cytoskeleton buffer for 10 min. Samples were quenched with 0.1% NaBH_4_ (SIGMA-Aldrich, St. Louis, MO) in 2.5 mL of freshly prepared PBS for 7 min on ice to reduce autofluorescence. Finally, fixed cells were washed with PBS twice, each time for a 10-min incubation, and then kept in PBS overnight at 4°C.

### TIRF 3D-N-STORM imaging of F-actin

Before imaging, F-actin was probed using 0.33 μM of Alexa Flour 647-conjugated phalloidin (Life Technologies, Waltham, MA) and incubated for 30 min at room temperature. The samples were imaged on a Nikon N-STORM microscope (Nikon, Tokyo, Japan) in TIRF mode. The microscope is equipped with a 100× NA 1.49 Apo TIRF objective lens, a back-illuminated EMCCD camera (Andor Ixon3, Belfast, UK), a Cy5 (excitation, 620/60; emission, 700/75) filter set (Chroma, Bellows Falls, VT), and a cylindrical lens for 3D imaging. During imaging, a 100-mW 641-nm laser (Coherent, Santa Clara, CA) and a 100-mW 405-nm laser (Coherent, Santa Clara, CA) were used to excite and photoswitch Alexa Fluor 647, respectively. In each image acquisition, a 5–10-min period of full-powered illumination by 641-nm laser was first performed to turn most Alexa Fluor 647 fluorophores into dark state to achieve a sparse distribution of single molecules. Image frames were acquired when single-molecule blinking could be observed. The intensity of 405-nm laser was periodically tuned during imaging to reactivate fluorophores to maintain sufficient fluorophore density.

For each image, 40,000 raw frames were acquired in the frame-transfer mode, with an exposure time of 50 ms, EM gain of 200, and a readout speed of 10 MHz. Fluorophore detection and localization were carried out by PeakSelector (courtesy of Harald Hess, Howard Hughes Medical Institute), a customized software developed in IDL (Exelis Vis, Boulder, CO). The centroid position of each detected fluorophore was calculated via 2D-Gaussian nonlinear least-square fitting, as described earlier [55]. The localization precision of each centroid coordinate was computed using the Thompson-Webb formula depicted earlier [56]. Fluorophore peaks with localization precisions smaller than 20 nm were rejected from subsequent analysis. The fiducials pre-embedded on the glass coverslips were used to correct drift. The N-STORM images were then reconstructed by representing each localization coordinate as a normalized Gaussian function whose widths depend on the localization precision [57]. Three-dimensional data were rendered with colors encoding the z positions of fluorophores.

### Measurement of F-actin meshwork occupancy

The N-STORM image of F-actin in Sertoli cells contains a mixture of fine meshwork and high-density bundles. To separate these two structures and analyze the density of the fine F-actin meshwork, the image was first reconstructed with a pixel size of 160 nm (an image size of 256 × 256 pixels) to blur regions of F-actin meshwork, leaving F-actin bundles as more prominent structures. The cell region was then anisotropically enhanced [58, 59] to highlight F-actin bundles, and then segmented using Otsu's method [60]. Next, another image was reconstructed with a pixel size of 10 nm, which is sufficiently high for the F-actin network, generating an image size of 4,096×4,096 pixels. The previously segmented regions of F-actin bundles were scaled up by 16 times and used as masks to isolate regions between thick F-actin bundles from the high-resolution image. The F-actin network was segmented from the isolated areas using Otsu's method. F-actin occupancy was then computed as the ratio between the number of F-actin pixels and the total number of pixels in the binarized regions of the F-actin network.

### Immunocytochemistry

For immunocytochemistry, cells were fixed with 4% PFA in PBS for 15 min at room temperature. The fixed cells were permeabilized with 0.1% Triton X-100 in PBS for 15 min at room temperature and then blocked with 5% skim milk in PBS for 1 h at room temperature. The cells were then stained with anti-pMLC antibodies (CST, Danvers, MA) and anti-espin1 antibodies (BD Biosciences, San Jose, CA). Secondary antibodies were Alexa Fluor 488-conjugated goat anti-mouse IgG (Molecular Probes, Eugene, OR) and Alexa Fluor 488-conjugated goat anti-rabbit IgG (Molecular Probes, Eugene, OR). F-actin was stained with Alexa Fluor 555-conjugated phalloidin (1:100; Molecular Probes, Eugene, OR). DAPI (Molecular Probes, Eugene, OR) was used for nuclear staining. Fluorescent images were acquired on a laser scanning confocal imaging system (Leica SP5) equipped with 100× NA 1.4 HCX PL APO CS oil immersion objective lens (Leica, Wetzlar, Germany). Images were processed using Adobe Photoshop CS5 software (Adobe, San Jose, CA).

### Measurement of pMLC intensity and proportion of epsin1-positive actin bundles

ROI including a cell was defined, and average pMLC intensity per pixel was then analyzed by ImageJ software. Averages of pMLC intensity per pixel of WT and *mDia1/3* DKO cells were generated based on the data obtained by ImageJ using Prism software (GraphPad Software, San Diego, CA). For quantification of the proportion of espin1-positive actin bundles, binary images of F-actin bundles and espin1 were generated by setting a threshold. The two images were then merged and the pixel number of total F-actin bundles and espin1 signals that are colocalized with F-actin bundles were analyzed. The proportion of espin1-positive F-actin bundles were then calculated as a ratio of the pixel number of espin1-associated F-actin bundles and total F-actin bundles for each cell. Averages of the proportion of espin1-positive actin bundles of WT and *mDia1/3* DKO cells were then generated using Prism software (GraphPad Software, San Diego, CA).

### Transfection and live imaging of primary cultured Sertoli cells

Primary cultured Sertoli cells were transfected with pCAG-*LifeAct-EGFP* [5] or pCAG-*EGFP-mDia3* [5] by electroporation using Neon Transfection System (Invitrogen, Carlsbad, CA) according to the manufacturer’s protocol. Briefly, 2×10^5^ cells were transfected with 1.5 μg of plasmid DNA. Electroporation was performed at a single pulse of 1,350 V for 30 ms. After electroporation, cells were plated in IMDM medium containing 10% FBS for 24 h in 5% CO_2_ at 37°C. For observation of LifeAct-EGFP, primary cultured Sertoli cells were maintained in IMDM without phenol red containing 2% FBS. Time-lapse imaging of LifeAct-EGFP was carried out at 37°C using a SD-OSR IX83 inverted microscope (Olympus, Tokyo, Japan) equipped with a 100× NA 1.4, UPLS APO oil immersion objective (Olympus, Tokyo, Japan) and Yokogawa W1 spinning disk unit (Yokogawa, Musashino, Japan) and controlled by MetaMorph software (Universal Imaging, San Jose, CA). An area near the cell periphery was selectively illuminated. Images were recorded at a rate of 2 s/frame. Speed, straightness, and frequency measurements were performed by tracking individual actin filaments manually using ImageJ software. Graphs were generated based on the data obtained by ImageJ using Prism software (GraphPad Software, San Diego, CA). Single-molecule speckle imaging of EGFP-mDia3 was acquired using a microscope (IX81, Olympus, Tokyo, Japan) equipped with 100-W mercury illumination, a Plan-Apo 100×, 1.4 NA oil-immersion objective (Olympus, Tokyo, Japan), and a cooled EMCCD camera (Evolve 512; Photometrics, Tucson, AZ). Cells expressing a low level of EGFP-mDia3 were observed. An area near the cell periphery was selectively illuminated. Images were recorded at a rate of 200 ms/frame. Measurements of speed, straightness, and travel distance of single-molecule movement were performed by tracking individual single molecules manually using ImageJ software. Graphs were generated based on the data obtained by ImageJ using Prism software (GraphPad Software, San Diego, CA). Kymograph analysis was performed using MetaMorph software (Universal Imaging, San Jose, CA).

### Germ cell isolation

Testes were isolated from the 3-wk-old *C57BL/6-Tg* (*CAG-EGFP*) mice, and the tunica and blood vessels were dissected and removed by fine forceps. Isolated testes were minced in PBS using scalpel blades. The minced tissue was then transferred into 50-mL plastic tubes and centrifuged at 100*g* for 1 min. Supernatant was discarded and the pellet was washed twice in PBS and centrifuged at 100*g* for 1 min to recover germ cells. Cells were then resuspended in PBS and filtered through the 40-μm nylon mesh to remove tissue debris and cell clumps. Germ cells were then centrifuged at 500*g* for 10 min. The resultant cell pellet was finally resuspended in Ham's F12/DMEM containing 10% FBS [61].

### Sertoli cell–germ cell cocultures

To remove residual germ cells contaminated in the primary culture of Sertoli cells prepared as described above, the primary cultures, after 3 d of incubation, were subjected to hypotonic treatment for 2 h at room temperature with 20 mM Tris-HCl buffer, pH 7.4, for 2 min [62]. Purified Sertoli cells were then incubated with 10% FBS in IMDM for 2 h in 5% CO_2_ at 37°C to recover from the hypotonic treatment. Germ cells (5×10^5^ cells/well) isolated from testes of 3-wk-old *C57BL/6-Tg* (*CAG-EGFP*) mice were then plated onto the Sertoli cells. These cells were cocultured for 1 d, fixed with 4% PFA/PBS for 15 min at room temperature, and subjected to immunocytochemistry. Fluorescent images were acquired on a SD-OSR IX83 inverted microscope (Olympus, Tokyo, Japan) equipped with a 100× NA 1.4, UPLS APO oil immersion objective (Olympus, Tokyo, Japan) and Yokogawa W1 spinning disk unit (Yokogawa, Musashino, Japan) controlled by MetaMorph software (Universal Imaging, San Jose, CA).

### Quantification analysis of stained Sertoli cell–germ cell cocultures

Living GFP-positive germ cells with round nuclear shape, as determined by DAPI staining and continuous N-cadherin signal around the germ cells, were defined as adhesive cells. Living GFP-positive germ cells with round nuclear shape, as determined by DAPI staining and continuous phalloidin staining surrounding the germ cells, were defined as germ cells with continuous F-actin cup. Cortical F-actin meshwork density and the continuity of the F-actin cup were quantified manually using ImageJ software. Graphs, including the plot of correlation, were generated based on the data obtained by ImageJ using Prism software (GraphPad Software, San Diego, CA).

### Scanning electron microscopy

Sperms prepared from the cauda epididymis and suspended as above were fixed with an equal volume of 2% PFA and 2% GA in 0.1 M cacodylate buffer. Thereafter, they were fixed with 1% GA in 0.1 M cacodylate buffer, pH 7.4, at 4°C overnight. The samples were further fixed with 1% tannic acid in 0.1 M cacodylate buffer, pH 7.4, at 4°C for 1 h. After the fixation, the samples were washed four times with 0.1 M cacodylate buffer for 30 min each, followed by postfixation with 2% OsO_4_ in 0.1 M cacodylate buffer at 4°C for 2 h. The samples were dehydrated through a series of graded ethanol (50%, 70%, 90%, 100%). The samples were substituted into tert-butyl alcohol at room temperature and then frozen. The frozen samples were vacuum dried. After drying, the samples were coated with a thin layer (30 nm) of osmium by using an osmium plasma coater (NL-OPC80NS, Nippon Laser & Electronics Laboratory, Nagoya, Japan). The samples were observed by a JSM-6340F scanning electron microscope (JEOL, Akishima, Japan) at an acceleration voltage of 5.0 kV.

### Ex vivo imaging of LifeAct-EGFP–labeled Sertoli cells

We constructed the pLV-*LifeAct-EGFP* (LV, lentivirus) by inserting a *LifeAct-EGFP* fragment amplified by PCR from pCAG- *LifeAct-EGFP* [5] with primers *LifeAct-EGFP* forward (5′-GCTCTAGAATGGGCGTGGCCGACCTGAT-3′) and *LifeAct-EGFP* reverse (5′-CTCTCGAGTTACTTGTACAGCTCGTCCATGCC-3′) into pLV-CAG1.1 (a gift from Dr. Inder Verma, Salk Institute). Lentivirus were generated as previously reported [32]. We injected recombinant lentivirus vectors into male C57BL6/N adult mice at 6 wk of age. Mice were anesthetized by i.p. injection of Avertin before the operation. Approximately 10 μL of lentivirus vector solution containing 0.04% trypan blue was injected into the right seminiferous tubules via the efferent ductules, according to the method described, and Sertoli cells were selectively labeled [32]. After 1 wk, mice were humanely killed and the injected right testis was dissected and the tunica albuginea were removed. Preparation of seminiferous tubules for imaging was then performed according the previous report [33], with slight modifications. Seminiferous tubules were disentangled and then fixed with 4% PFA for 1 h at room temperature. Fixed seminiferous tubules were then cut into small pieces and attached to MAS-coated slide glass (Matsunami, Kishiwada, Japan) by half-drying. Specimens were mounted in antifade prolong diamond (Thermo Fisher Scientific, Waltham, MA). Samples were observed under SD-OSR IX83 inverted microscope (Olympus, Tokyo, Japan) equipped with a 100× NA 1.4, UPLS APO silicone immersion objective (Olympus, Tokyo, Japan), and Yokogawa W1 spinning disk unit (Yokogawa, Musashino, Japan) and controlled by MetaMorph software (Universal Imaging, San Jose, CA). Deconvolution processing of stacked images was performed by cellSens Dimension software (Olympus, Tokyo, Japan). Three-dimensional reconstruction of stacked images was generated by Volocity software (PerkinElmer, Waltham, MA).

### Statistical analysis

Prism (GraphPad Software, San Diego, CA) and Excel (Microsoft, Redmond, WA) were used for statistical analyses. Data are presented as mean ± SEM, and were analyzed by one-way factorial ANOVA or unpaired Student *t* test. *P* < 0.05 was considered statistically significant.

## Supporting information

S1 Fig Male infertility of *mDia1/3* DKO mice. (A and B) Micrographs (A) and fertilization rate (B) of IVF of ZP-intact oocytes with sperms from WT or *mDia1/3* DKO mice. Scale bar, 50 μm. Data represented mean ± SEM. Data are the average of three independent experiments. ***P < 0.001 (Student *t* test). DKO, double knockout; IVF, in vitro fertilization; *mDia1/3*, mammalian diaphanous homolog1/3; WT, wild-type; ZP, Zona pellucida.
(TIF)

S2 Fig Reduced number, abnormal morphology, and impaired motility of *mDia1/3* DKO sperm. (A) HE-stained epididymal cross sections from adult WT or *mDia1/3* DKO mice. Scale bar, 25 μm. (B) Total number of sperm per epididymis of WT or *mDia1/3* DKO mice. Data represented mean ± SEM. *n* = 3 and 4 for WT and *mDia1/3* DKO, respectively. **P < 0.01 (*P* = 0.0062, Student *t* test). (C) Bright-field micrographs of sperm isolated from cauda epididymis. Note that *mDia1/3* DKO spermatozoa exhibited abnormal head (arrowhead) and tail (arrow) morphology. Scale bar, 10 μm. (D) SEM micrographs of the head of WT and *mDia1/3* DKO sperm isolated from cauda epididymis. Note the abnormal shape of *mDia1/3* DKO sperm head. Scale bar, 1 μm. (E) WT and *mDia1/3* DKO sperm motility at 0 and 3 h after sperm suspension. Data represented mean ± SEM. *n* = 3 for each genotype. *P < 0.05, **P < 0.01 (*P* = 0.003 for 0 h and *P* = 0.0111 for 3 h, Student *t* test). (F) A cartoon depicted different parameters for sperm motility, determined by CASA. (G) Quantification of VAP (average path velocity) of sperm motility from WT (black) and *mDia1/3* DKO (white) sperms isolated from cauda epididymis at 0 and 3 h after sperm suspension. Data represented mean ± SEM. *n* = 3 for each genotype. *P < 0.05 (*P* = 0.0263 for 0 h and *P* = 0.0138 for 3 h, Student *t* test). (H) Quantification of VSL (straight-line velocity) of sperm motility from WT (black) and *mDia1/3* DKO (white) sperms isolated from cauda epididymis at 0 and 3 h after sperm suspension. Data represented mean ± SEM. *n* = 3 for each genotype. *P < 0.05 (*P* = 0.1569 for 0 h and *P* = 0.0251 for 3 h, Student *t* test). (I) Quantification of VCL (curvilinear velocity) of sperm motility from WT (black) and *mDia1/3* DKO (white) sperms isolated from cauda epididymis at 0 and 3 h after sperm suspension. Data represented mean ± SEM. *n* = 3 for each genotype. *P < 0.05 (*P* = 0.0177 for 0 h and P = 0.0157 for 3 h, Student *t* test). CASA, computer-assisted sperm analysis; DKO, double knockout; HE, hematoxylin–eosin; *mDia1/3*, mammalian diaphanous homolog1/3; n.s., not significant; VAP, average path velocity; VCL, curvilinear velocity; VSL, straight-line velocity; WT, wild-type.
(TIF)

S3 Fig Increased apoptotic cells in *mDia1/3* DKO seminiferous tubule. (A) Apoptotic cells (green) in the *mDia1/3* DKO seminiferous tubules. Nuclei (magenta) were stained with Hoechst. Scale bar, 100 μm. (B) Quantification of the number of apoptotic cells per seminiferous tubule. Data represented mean ± SEM (91 seminiferous tubules from four WT mice and 99 seminiferous tubules from four *mDia1/3* DKO mice). ***P < 0.001 (Student *t* test). DKO, double knockout; *mDia1/3*, mammalian diaphanous homolog1/3; WT, wild-type.
(TIF)

S4 Fig Specificity of anti-mDia1 and anti-mDia3 antibodies for immunohistochemistry. (A) Immunohistochemistry staining for mDia1 (green) and vimentin (magenta) as a marker for Sertoli cells in testis sections from WT and *mDia1* KO adult mice. Positive mDia1 signals at the vimentin-positive Sertoli cells observed in WT mice were abolished in *mDia1* KO mice. Scale bar, 100 μm. (B) Immunohistochemistry staining for mDia3 (green) and vimentin (magenta) as a marker for Sertoli cells in testis sections from WT and *mDia3* KO adult mice. Positive mDia3 signals at the vimentin-positive Sertoli cells observed in WT mice were abolished in *mDia3* KO mice. Scale bar, 100 μm. KO, knockout; mDia1, mammalian diaphanous homolog1; mDia3, mammalian diaphanous homolog3; WT, wild-type.
(TIF)

S5 Fig mDia3 expression in the seminiferous tubules throughout the spermatogenic cycles. (A) Immunohistochemistry staining for mDia3 (green) and phalloidin staining (magenta) of WT testis sections. Arrowheads indicate mDia3 staining at the basal ectoplasmic junction and arrows indicate mDia3 staining at the apical ectoplasmic junction. (B) Immunohistochemistry staining for mDia3 (green) and phalloidin staining (magenta) of *mDia3* KO testis sections. Positive mDia3 signals observed in WT mice were mostly abolished in *mDia3* KO seminiferous tubules, confirming the specificity of mDia3 antibodies. White asterisks indicate nonspecific staining signals in Leydig cells. Scale bars, 100 μm. KO, knockout; mDia3, mammalian diaphanous homolog3; WT, wild-type.
(TIF)

S6 Fig Reduced F-actin staining of *mDia1/3* DKO primary cultured Sertoli cell. (A) Confocal images of actin filaments of WT (left) and *mDia1/3* DKO (right) primary cultured Sertoli cells. The lines (magenta and green) were used to quantify the fluorescence intensity by line scan, and the fluorescence intensity profiles along these lines are shown in the right. Scale bar, 20 μm. DKO, double knockout; F-actin, filamentous actin; *mDia1/3*, mammalian diaphanous homolog1/3; WT, wild-type.
(TIF)

S7 Fig Reduced cortical F-actin meshwork in *mDia1/3* DKO Sertoli cells was rescued by expression of *EGFP-mDia3*. (A) *mDia1/3* DKO primary cultured Sertoli cells transfected with p*EGFP-mDia3* (green) were stained with phalloidin (magenta). The cell on the right is EGFP-mDia3 positive. The magenta and green dotted line was used to quantify the fluorescence intensity of *mDia1/3* DKO Sertoli cells and EGFP-mDia3 expressed *mDia1/3* DKO Sertoli cells in the line scan, subsequently. Scale bar, 50 μm. (B) Fluorescence intensity profiles along the line shown in S7A Fig. (C) Quantification of average normalized F-actin intensity per cell. Data represented mean ± SEM. Data are a sum of three independent experiments. *n* = 19 and 20 for *mDia1/3* DKO and *mDia1/3* DKO + *EGFP-mDia3* Sertoli cells, respectively. ***P < 0.001 (Student *t* test). DKO, double knockout; *EGFP*, enhanced green fluorescent protein; F-actin, filamentous actin; *mDia1/3*, mammalian diaphanous homolog1/3; p*EGFP*, enhanced green fluorescent protein expression plasmid.
(TIF)

S8 Fig F-actin architecture of Sertoli cells in intact seminiferous tubules. (A) Transduction of *LifeAct-EGFP* expressing lentivirus in the testis of WT mouse. Seminiferous tubule was microinjected with *LifeAct-EGFP* expressing lentivirus and analyzed at 1 wk after injection under fluorescence stereomicroscope. (B) Observation strategy of *LifeAct-EGFP* expressing lentivirus transduced Sertoli cells. To observe LifeAct-EGFP of Sertoli cells in intact seminiferous tubules ex vivo, seminiferous tubules of the microinjected testis were dissected and untangled under fluorescence stereomicroscope. Isolated seminiferous tubules were then cut into small pieces about 2–3 mm, fixed, and then mounted between a slide glass and cover glass. The LifeAct-EGFP–labeled Sertoli cell near the cover glass was then observed with an inverted confocal microscope equipped with a spinning disk through the peritubular myoid cell layer and basal membrane. (C) A stacked spinning disk confocal image of a *LifeAct-EGFP* expressing lentivirus transduced Sertoli cell. Image was processed with a deconvolution algorithm. Scale bar, 10 μm. The red and blue boxes are magnified images of the corresponding boxed areas of the lower magnification. Arrowhead indicates thick F-actin bundles and arrows indicate fine meshwork F-actin filaments. *EGFP*, enhanced green fluorescent protein; F-actin, filamentous actin; WT, wild-type.
(TIF)

S9 Fig Nectin-2 expression in the seminiferous tubules throughout the spermatogenic cycles. (A) Immunohistochemistry staining for nectin-2 (green) and Hoechst staining (magenta) of testis sections from WT mice. Strong nectin-2 signals were observed at the apical ectoplasmic specialization junction. In addition, nectin-2 signals were observed at the adherens junction located on the boundary between cells. (B) Immunohistochemistry staining for nectin-2 (green) and Hoechst staining (magenta) of testis sections from *mDia1/3* DKO mice. Nectin-2 signals at both the apical ectoplasmic specialization junction and adherens junction were reduced. Scale bars, 100 μm. DKO, double knockout; *mDia1/3*, mammalian diaphanous homolog1/3; WT, wild-type.
(TIF)

S10 Fig Immunohistochemistry of espin1 together with phalloidin staining in seminiferous tubule throughout spermatogenic cycles. (A) Immunohistochemistry staining for espin1 (green) and phalloidin staining (magenta) of testis sections from WT mice. Espin1 signals were observed at the apical ES junction of elongated spermatid from stages II–V seminiferous tubules of the WT mice. (B) Immunohistochemistry staining for espin1 (green) and phalloidin staining (magenta) of testis sections from *mDia1/3* DKO mice. Espin1 signals were ectopically localized to the abnormal F-actin bundles in the process of Sertoli cells throughout the spermatogenic cycle. Scale bars, 100 μm. DKO, double knockout; ES, ectoplasmic specialization; F-actin, filamentous actin; *mDia1/3*, mammalian diaphanous homolog1/3; WT, wild-type.
(TIF)

S11 Fig Model of the molecular mechanism of mDia1/3 function in spermatogenesis through the regulation of F-actin and cell adhesions between Sertoli cell and germ cell. F-actin, filamentous actin; mDia1/3, mammalian diaphanous homolog1/3.
(TIF)

S1 Movie F-actin dynamics observed with SDSRM. *EGFP-LifeAct* was expressed in primary cultured Sertoli cell from WT mouse; the images were taken every 2 s. *EGFP*, enhanced green fluorescent protein; F-actin, filamentous actin; SDSRM, spinning disk superresolution microscopy; WT, wild-type.
(MOV)

S2 Movie Fast polymerization of cortical actin filament meshwork observed with SDSRM. *EGFP-LifeAct* was expressed in primary cultured Sertoli cell from WT mouse; the images were taken every 2 s. *EGFP*, enhanced green fluorescent protein; SDSRM, spinning disk superresolution microscopy; WT, wild-type.
(MOV)

S3 Movie Intracellular molecular movement of EGFP-mDia3 underneath the cell cortex, observed with TIRF microscopy. EGFP-mDia3 was expressed in primary cultured Sertoli cell from WT mouse; the images were taken every 200 ms. EGFP, enhanced green fluorescent protein; mDia3, mammalian diaphanous homolog3; TIRF, total internal reflection; WT, wild-type.
(MOV)

S4 Movie Single-molecule observation of fast movement of EGFP-mDia3 in a primary cultured Sertoli cell with TIRF microscopy. EGFP-mDia3 was expressed in primary cultured Sertoli cell from WT mouse; the images were taken every 200 ms. EGFP, enhanced green fluorescent protein; mDia3, mammalian diaphanous homolog3; TIRF, total internal reflection; WT, wild-type.
(MOV)

S5 Movie Three-dimensional reconstruction of deconvolution spinning disk confocal images of Sertoli cell F-actin in intact seminiferous tubule. Z-series images of Sertoli cell transduced by *LifeAct-EGFP* expressing lentivirus in intact seminiferous tubule were acquired by spinning disk confocal microscopy and processed with a deconvolution algorithm. Three-dimensional reconstruction and movie were generated by Volocity software. *EGFP*, enhanced green fluorescent protein; F-actin, filamentous actin.
(MOV)

## Abbreviations

3D: three dimensional
ABP: actin binding protein
acro/act-EGFP: *acrosin/actin-*enhanced green fluorescent protein
AJ: adherens junction
Arp2/3: actin-related protein2/3
a.u.: arbitrary unit
DKO: double knockout
eCG: equine chorionic gonadotropin
EGFP: enhanced green fluorescent protein
ES: ectoplasmic specialization
F-actin: filamentous actin
G-actin: globular actin
GFP: green fluorescent protein
HBSS: Hanks’ balanced salt solution
hCG: human chorionic gonadotropin
HE: hematoxylin–eosin
IMDM: Iscove’s modified Dulbecco’s medium
IVF: in vitro fertilization
KO: knockout
mDia: mammalian diaphanous homolog
N-cad: neural cadherin
N-cadherin: neural cadherin
PAS: Periodic acid-Schiff
p*EGFP*: enhanced green fluorescent protein expression plasmid
pMLC: phosphorylated myosin light chain
PNA: peanut agglutinin
ROI: region of interest
SDSRM: spinning disk superresolution microscopy
SMIFH2: small molecule inhibitor of formin homology 2 domain
STORM: stochastic optical reconstruction microscopy
TIRF: total internal reflection
VAP: average path velocity
VCL: curvilinear velocity
VSL: straight-line velocity
WT: wild-type
