# Supplementary figures and images for: mDia1/3 generate cortical F-actin meshwork in Sertoli cells that is continuous with contractile F-actin bundles and indispensable for spermatogenesis and male fertility
Source: PLoS Biol. 2018 Sep 26;16(9):e2004874. doi: 10.1371/journal.pbio.2004874 (PMC6175529; doi:10.1371/journal.pbio.2004874)

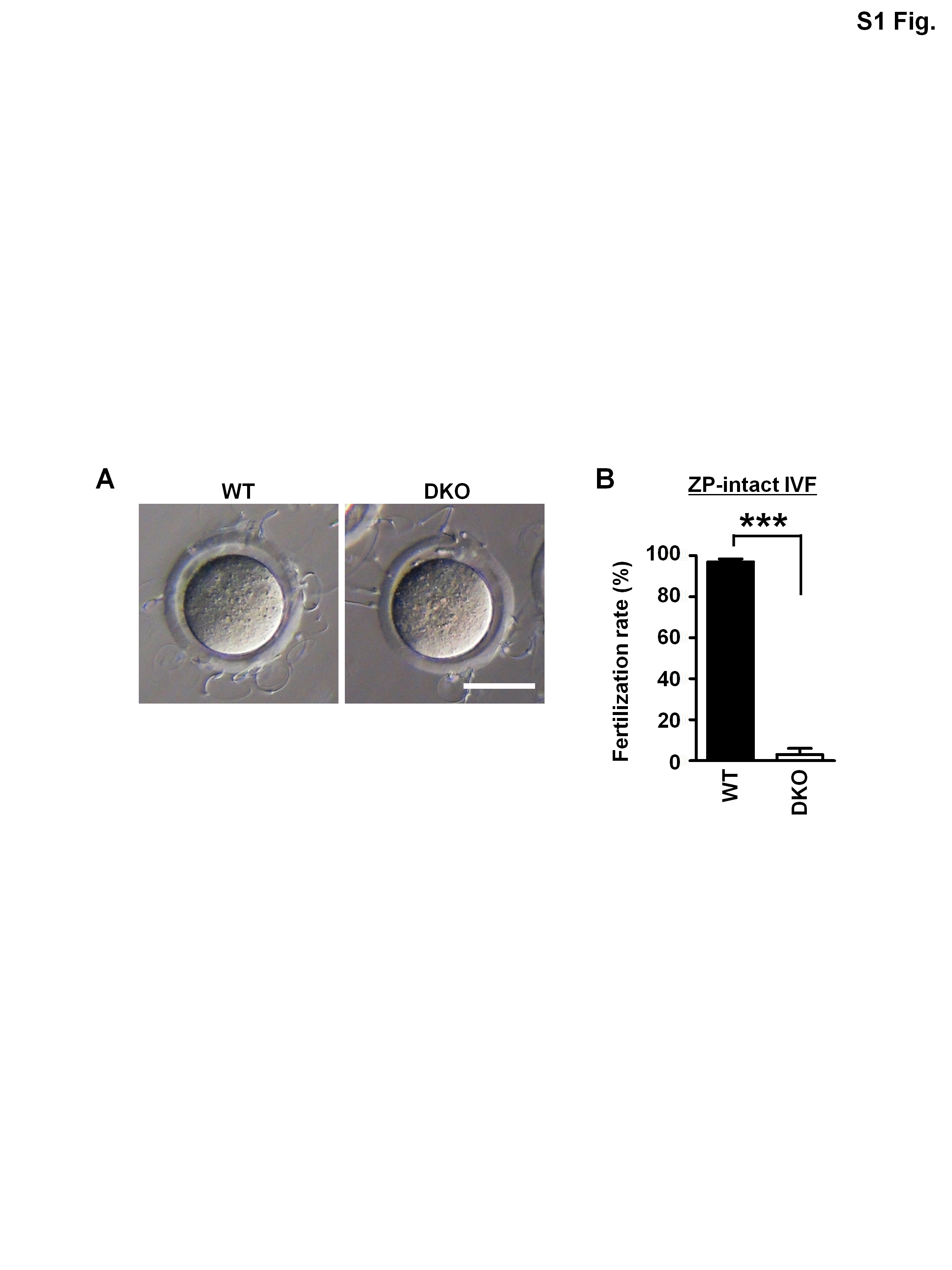

Supplement: S1 Fig — (A and B) Micrographs (A) and fertilization rate (B) of IVF of ZP-intact oocytes with sperms from WT or mDia1/3 DKO mice. Scale bar, 50 μm. Data represented mean ± SEM. Data are the average of three independent experiments. ***P < 0.001 (Student t test). DKO, double knockout; IVF, in vitro fertilization; mDia1/3, mammalian diaphanous homolog1/3; WT, wild-type; ZP, Zona pellucida. (TIF) [file pbio.2004874.s001.tif]

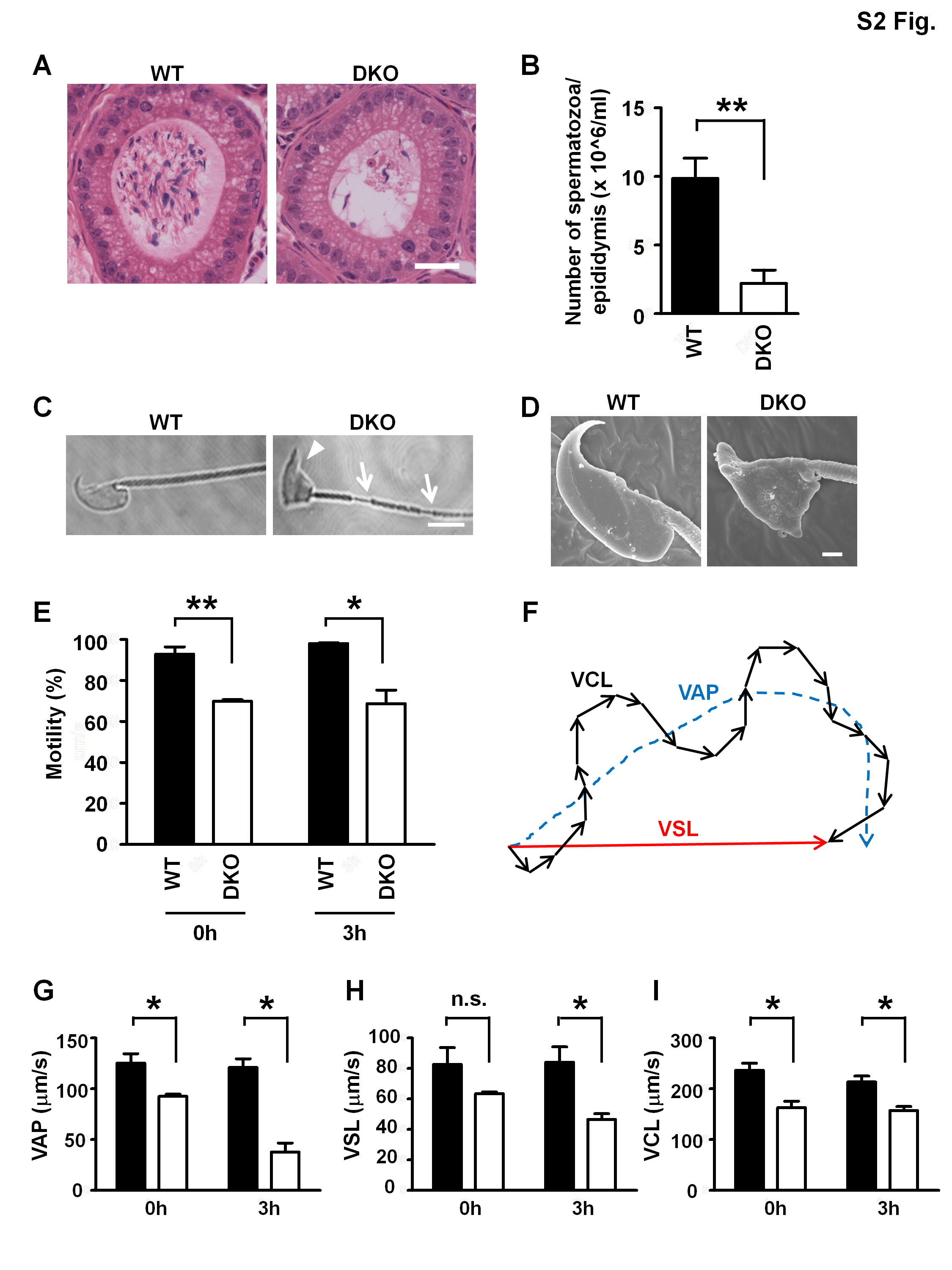

Supplement: S2 Fig — (A) HE-stained epididymal cross sections from adult WT or mDia1/3 DKO mice. Scale bar, 25 μm. (B) Total number of sperm per epididymis of WT or mDia1/3 DKO mice. Data represented mean ± SEM. n = 3 and 4 for WT and mDia1/3 DKO, respectively. **P < 0.01 (P = 0.0062, Student t test). (C) Bright-field micrographs of sperm isolated from cauda epididymis. Note that mDia1/3 DKO spermatozoa exhibited abnormal head (arrowhead) and tail (arrow) morphology. Scale bar, 10 μm. (D) SEM micrographs of the head of WT and mDia1/3 DKO sperm isolated from cauda epididymis. Note the abnormal shape of mDia1/3 DKO sperm head. Scale bar, 1 μm. (E) WT and mDia1/3 DKO sperm motility at 0 and 3 h after sperm suspension. Data represented mean ± SEM. n = 3 for each genotype. *P < 0.05, **P < 0.01 (P = 0.003 for 0 h and P = 0.0111 for 3 h, Student t test). (F) A cartoon depicted different parameters for sperm motility, determined by CASA. (G) Quantification of VAP (average path velocity) of sperm motility from WT (black) and mDia1/3 DKO (white) sperms isolated from cauda epididymis at 0 and 3 h after sperm suspension. Data represented mean ± SEM. n = 3 for each genotype. *P < 0.05 (P = 0.0263 for 0 h and P = 0.0138 for 3 h, Student t test). (H) Quantification of VSL (straight-line velocity) of sperm motility from WT (black) and mDia1/3 DKO (white) sperms isolated from cauda epididymis at 0 and 3 h after sperm suspension. Data represented mean ± SEM. n = 3 for each genotype. *P < 0.05 (P = 0.1569 for 0 h and P = 0.0251 for 3 h, Student t test). (I) Quantification of VCL (curvilinear velocity) of sperm motility from WT (black) and mDia1/3 DKO (white) sperms isolated from cauda epididymis at 0 and 3 h after sperm suspension. Data represented mean ± SEM. n = 3 for each genotype. *P < 0.05 (P = 0.0177 for 0 h and P = 0.0157 for 3 h, Student t test). CASA, computer-assisted sperm analysis; DKO, double knockout; HE, hematoxylin–eosin; mDia1/3, mammalian diaphanous homolog1/3; n.s., not significant; VA [file pbio.2004874.s002.tif]

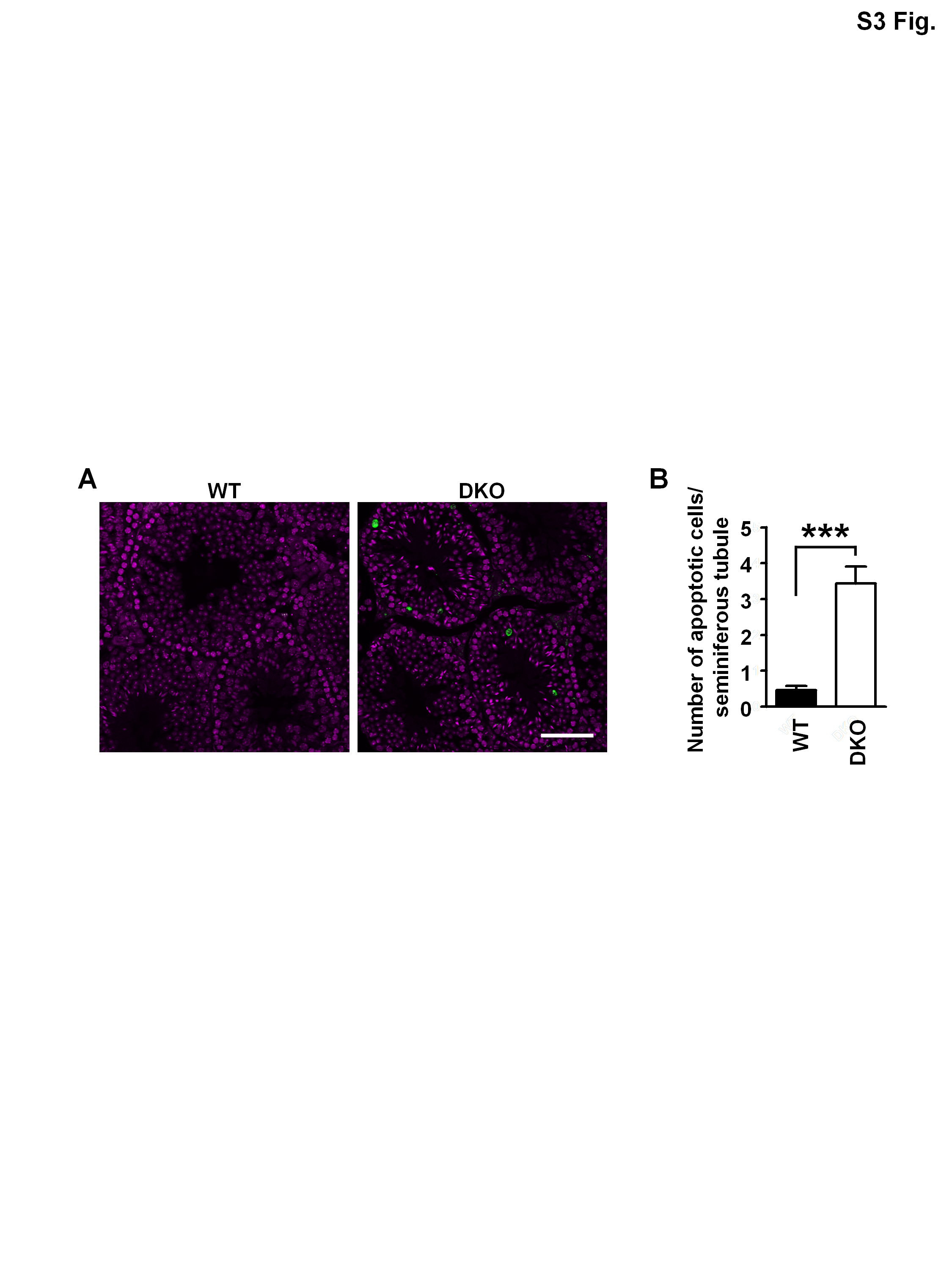

Supplement: S3 Fig — (A) Apoptotic cells (green) in the mDia1/3 DKO seminiferous tubules. Nuclei (magenta) were stained with Hoechst. Scale bar, 100 μm. (B) Quantification of the number of apoptotic cells per seminiferous tubule. Data represented mean ± SEM (91 seminiferous tubules from four WT mice and 99 seminiferous tubules from four mDia1/3 DKO mice). ***P < 0.001 (Student t test). DKO, double knockout; mDia1/3, mammalian diaphanous homolog1/3; WT, wild-type. (TIF) [file pbio.2004874.s003.tif]

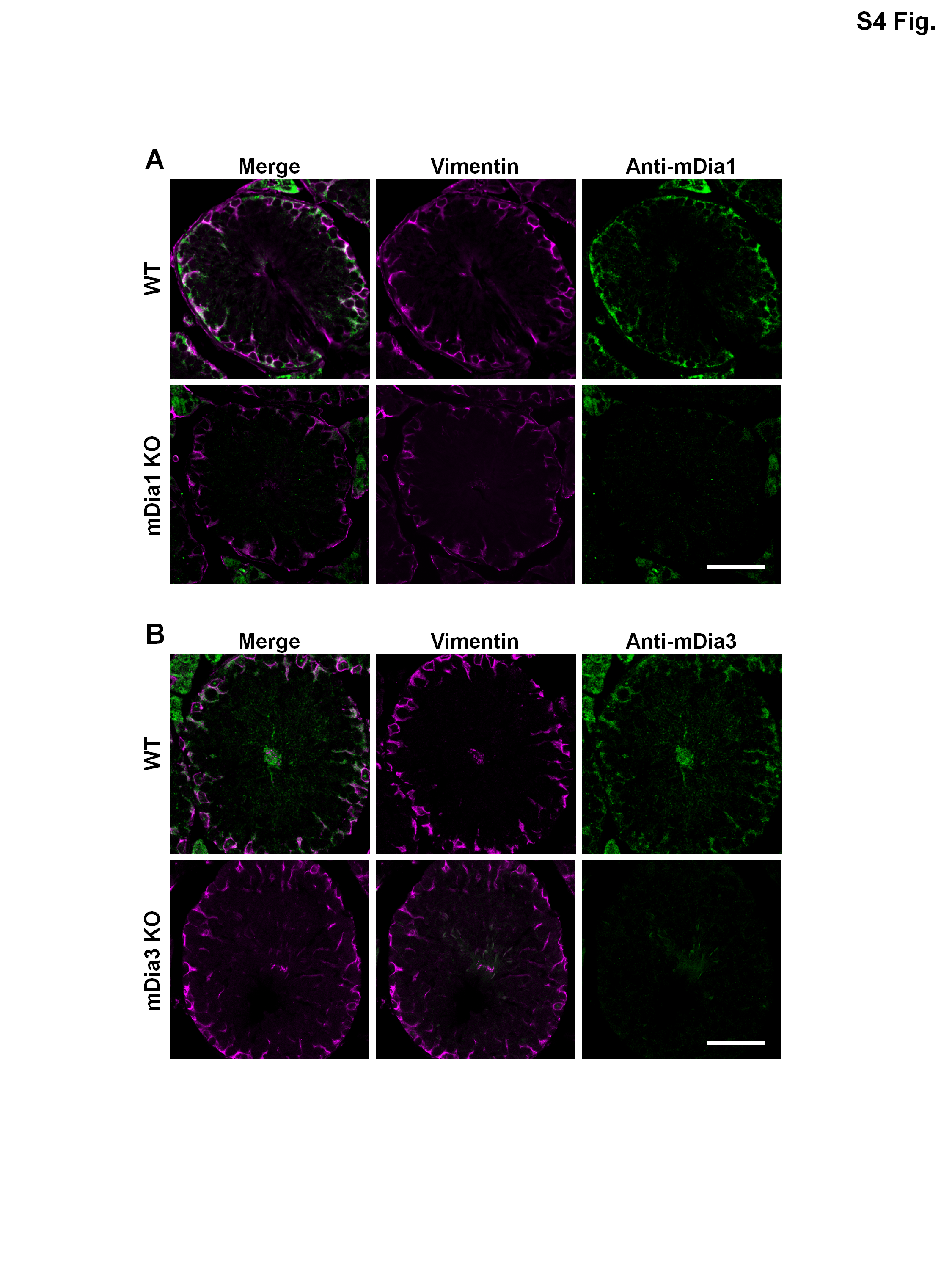

Supplement: S4 Fig — (A) Immunohistochemistry staining for mDia1 (green) and vimentin (magenta) as a marker for Sertoli cells in testis sections from WT and mDia1 KO adult mice. Positive mDia1 signals at the vimentin-positive Sertoli cells observed in WT mice were abolished in mDia1 KO mice. Scale bar, 100 μm. (B) Immunohistochemistry staining for mDia3 (green) and vimentin (magenta) as a marker for Sertoli cells in testis sections from WT and mDia3 KO adult mice. Positive mDia3 signals at the vimentin-positive Sertoli cells observed in WT mice were abolished in mDia3 KO mice. Scale bar, 100 μm. KO, knockout; mDia1, mammalian diaphanous homolog1; mDia3, mammalian diaphanous homolog3; WT, wild-type. (TIF) [file pbio.2004874.s004.tif]

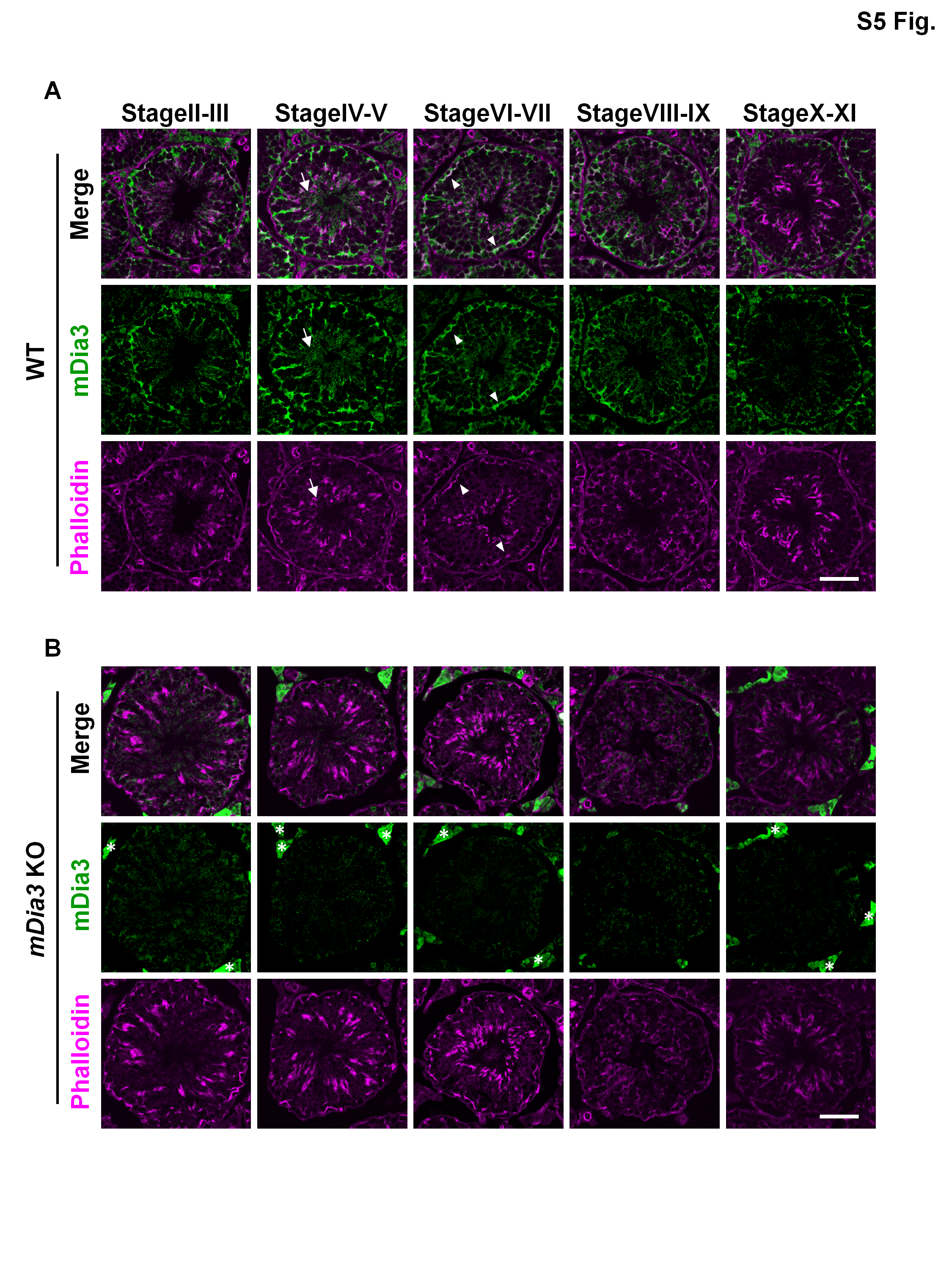

Supplement: S5 Fig — (A) Immunohistochemistry staining for mDia3 (green) and phalloidin staining (magenta) of WT testis sections. Arrowheads indicate mDia3 staining at the basal ectoplasmic junction and arrows indicate mDia3 staining at the apical ectoplasmic junction. (B) Immunohistochemistry staining for mDia3 (green) and phalloidin staining (magenta) of mDia3 KO testis sections. Positive mDia3 signals observed in WT mice were mostly abolished in mDia3 KO seminiferous tubules, confirming the specificity of mDia3 antibodies. White asterisks indicate nonspecific staining signals in Leydig cells. Scale bars, 100 μm. KO, knockout; mDia3, mammalian diaphanous homolog3; WT, wild-type. (TIF) [file pbio.2004874.s005.tif]

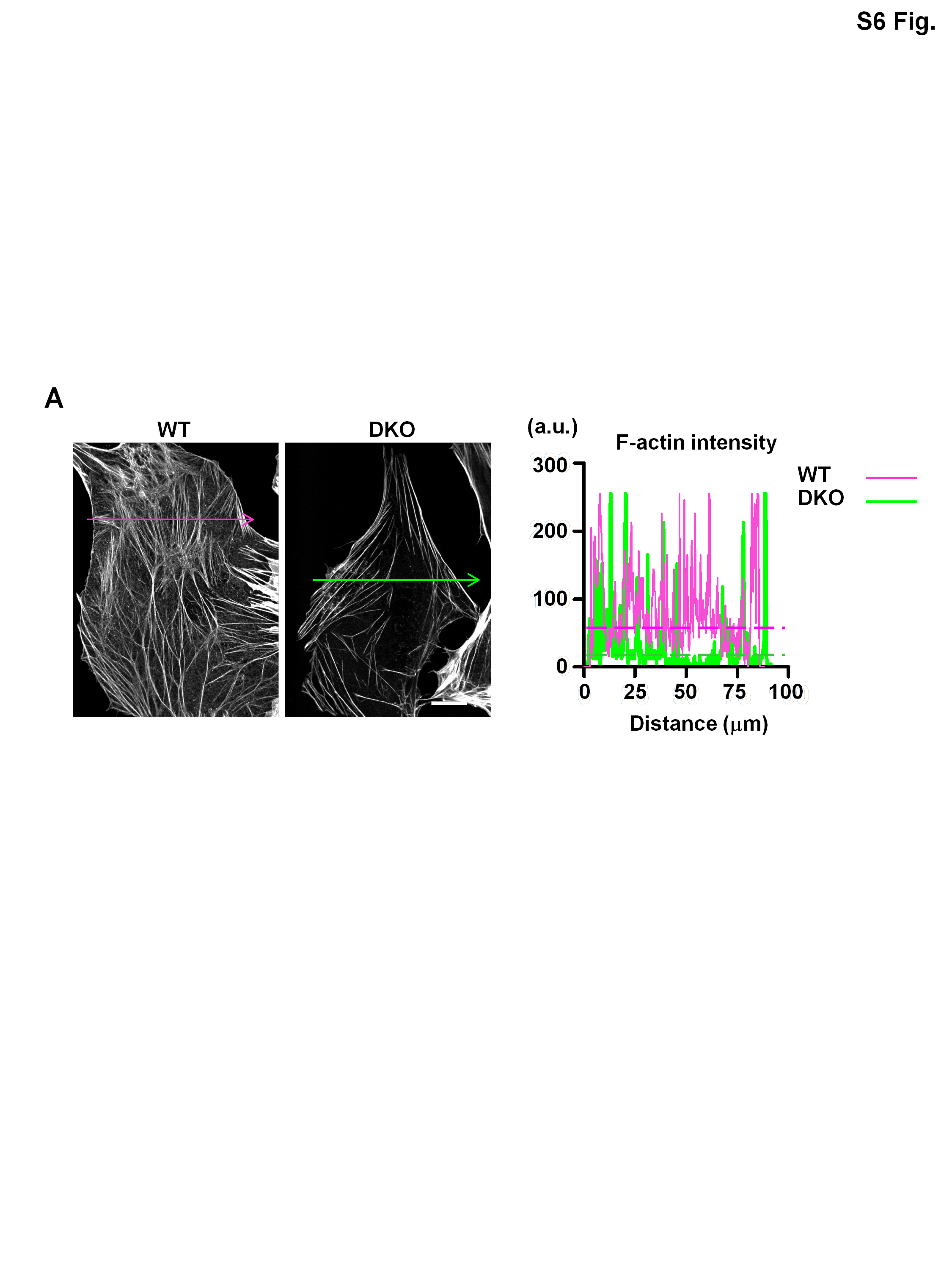

Supplement: S6 Fig — (A) Confocal images of actin filaments of WT (left) and mDia1/3 DKO (right) primary cultured Sertoli cells. The lines (magenta and green) were used to quantify the fluorescence intensity by line scan, and the fluorescence intensity profiles along these lines are shown in the right. Scale bar, 20 μm. DKO, double knockout; F-actin, filamentous actin; mDia1/3, mammalian diaphanous homolog1/3; WT, wild-type. (TIF) [file pbio.2004874.s006.tif]

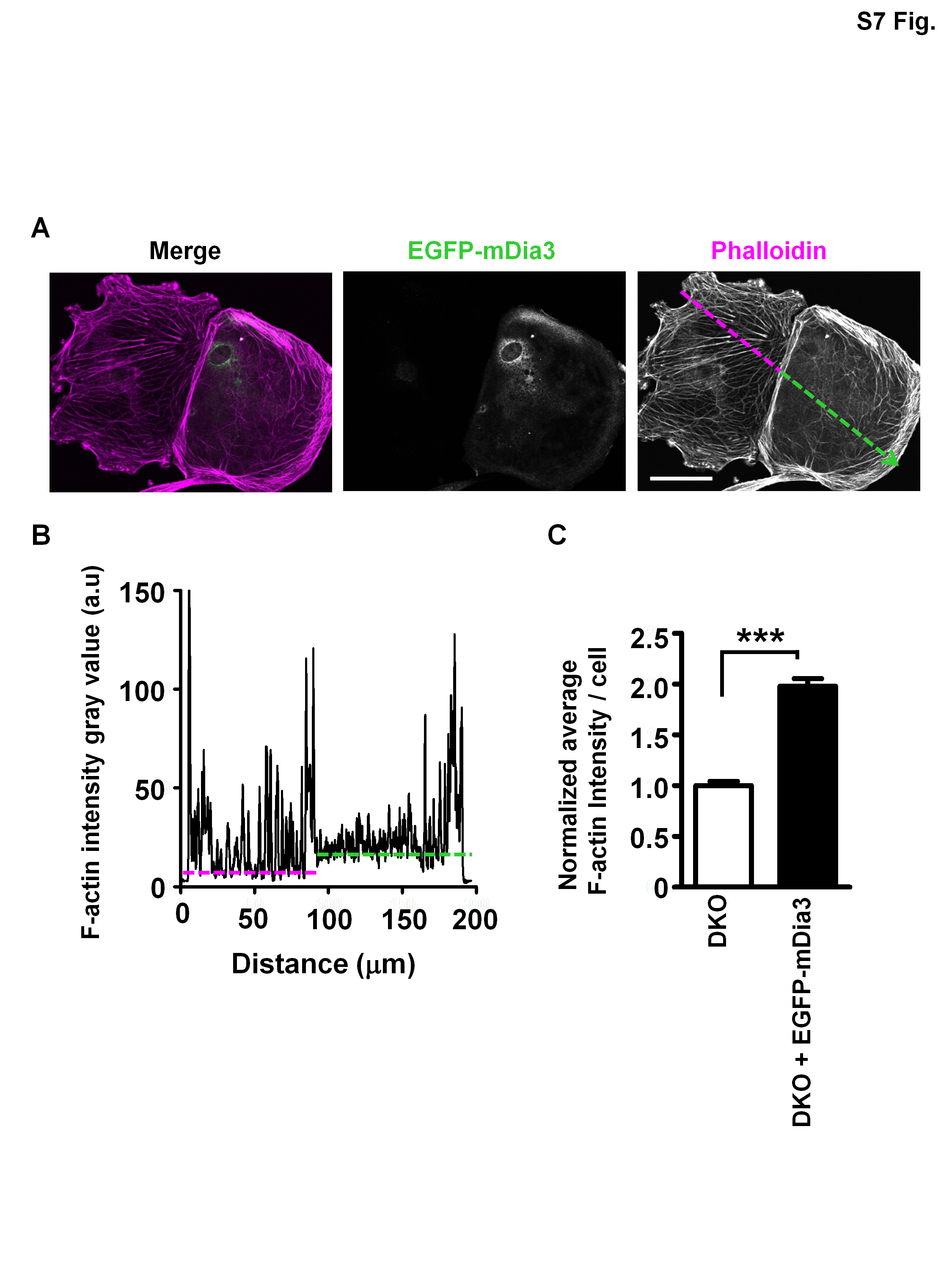

Supplement: S7 Fig — (A) mDia1/3 DKO primary cultured Sertoli cells transfected with pEGFP-mDia3 (green) were stained with phalloidin (magenta). The cell on the right is EGFP-mDia3 positive. The magenta and green dotted line was used to quantify the fluorescence intensity of mDia1/3 DKO Sertoli cells and EGFP-mDia3 expressed mDia1/3 DKO Sertoli cells in the line scan, subsequently. Scale bar, 50 μm. (B) Fluorescence intensity profiles along the line shown in S7A Fig. (C) Quantification of average normalized F-actin intensity per cell. Data represented mean ± SEM. Data are a sum of three independent experiments. n = 19 and 20 for mDia1/3 DKO and mDia1/3 DKO + EGFP-mDia3 Sertoli cells, respectively. ***P < 0.001 (Student t test). DKO, double knockout; EGFP, enhanced green fluorescent protein; F-actin, filamentous actin; mDia1/3, mammalian diaphanous homolog1/3; pEGFP, enhanced green fluorescent protein expression plasmid. (TIF) [file pbio.2004874.s007.tif]

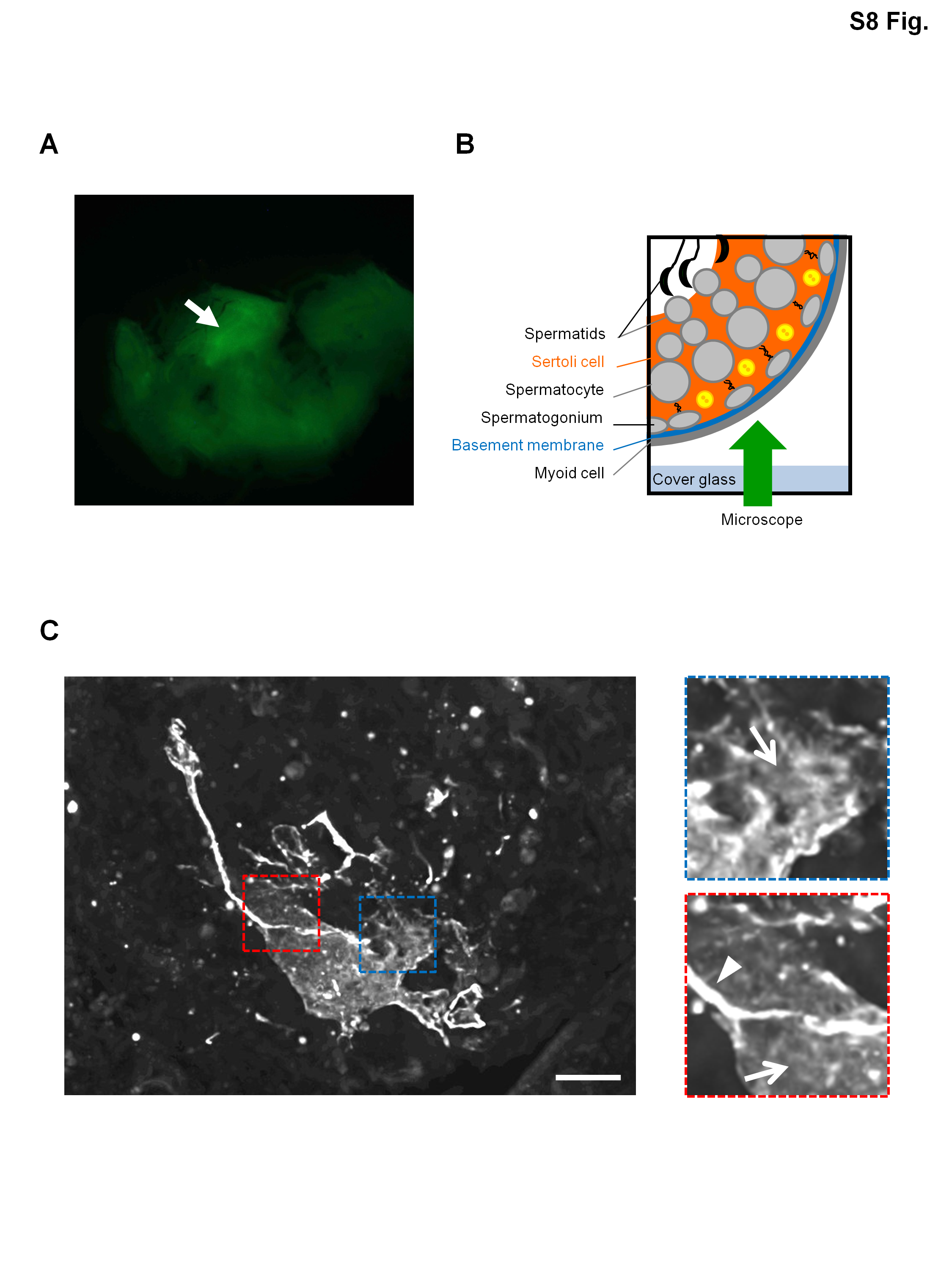

Supplement: S8 Fig — (A) Transduction of LifeAct-EGFP expressing lentivirus in the testis of WT mouse. Seminiferous tubule was microinjected with LifeAct-EGFP expressing lentivirus and analyzed at 1 wk after injection under fluorescence stereomicroscope. (B) Observation strategy of LifeAct-EGFP expressing lentivirus transduced Sertoli cells. To observe LifeAct-EGFP of Sertoli cells in intact seminiferous tubules ex vivo, seminiferous tubules of the microinjected testis were dissected and untangled under fluorescence stereomicroscope. Isolated seminiferous tubules were then cut into small pieces about 2–3 mm, fixed, and then mounted between a slide glass and cover glass. The LifeAct-EGFP–labeled Sertoli cell near the cover glass was then observed with an inverted confocal microscope equipped with a spinning disk through the peritubular myoid cell layer and basal membrane. (C) A stacked spinning disk confocal image of a LifeAct-EGFP expressing lentivirus transduced Sertoli cell. Image was processed with a deconvolution algorithm. Scale bar, 10 μm. The red and blue boxes are magnified images of the corresponding boxed areas of the lower magnification. Arrowhead indicates thick F-actin bundles and arrows indicate fine meshwork F-actin filaments. EGFP, enhanced green fluorescent protein; F-actin, filamentous actin; WT, wild-type. (TIF) [file pbio.2004874.s008.tif]

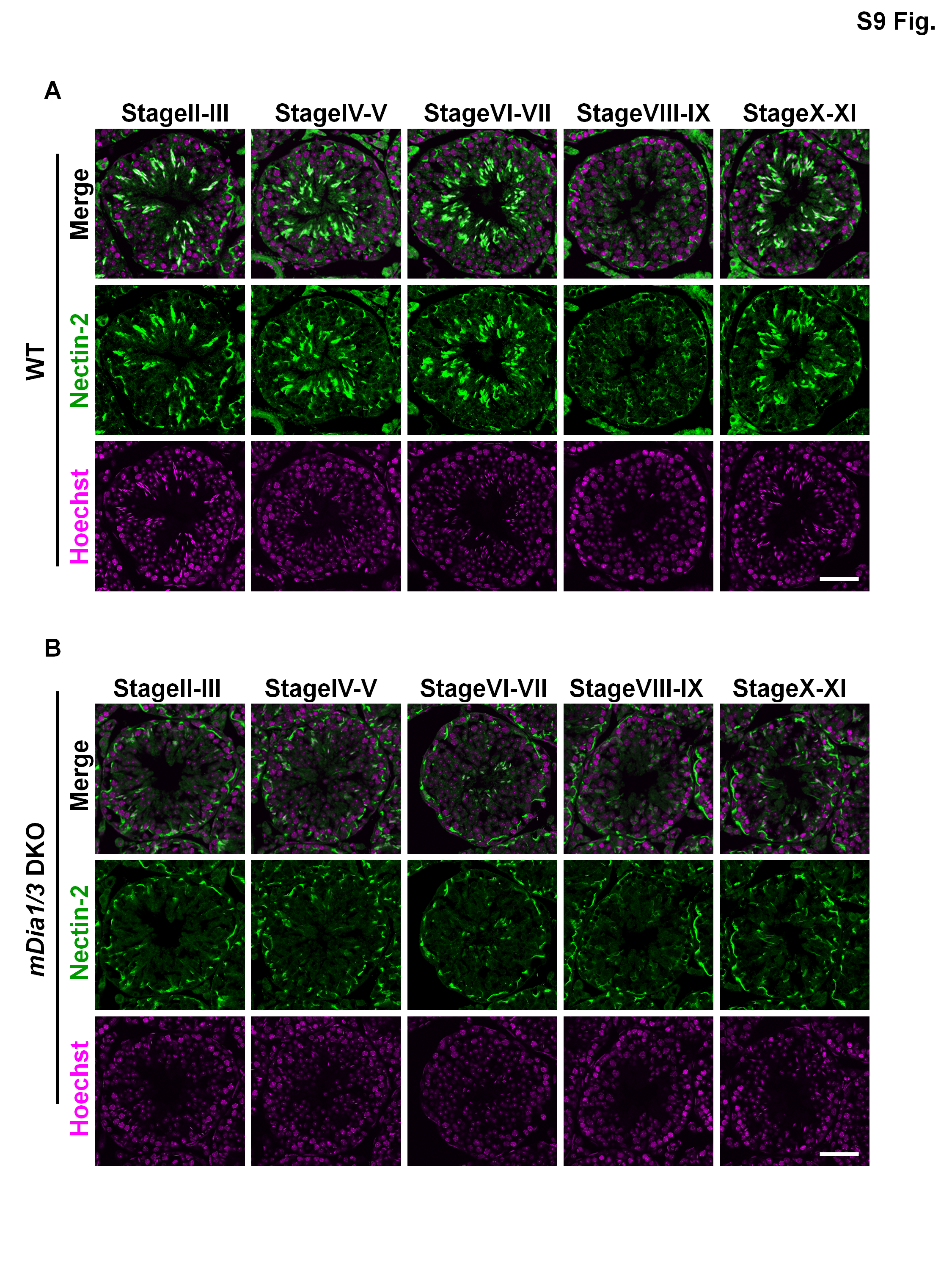

Supplement: S9 Fig — (A) Immunohistochemistry staining for nectin-2 (green) and Hoechst staining (magenta) of testis sections from WT mice. Strong nectin-2 signals were observed at the apical ectoplasmic specialization junction. In addition, nectin-2 signals were observed at the adherens junction located on the boundary between cells. (B) Immunohistochemistry staining for nectin-2 (green) and Hoechst staining (magenta) of testis sections from mDia1/3 DKO mice. Nectin-2 signals at both the apical ectoplasmic specialization junction and adherens junction were reduced. Scale bars, 100 μm. DKO, double knockout; mDia1/3, mammalian diaphanous homolog1/3; WT, wild-type. (TIF) [file pbio.2004874.s009.tif]

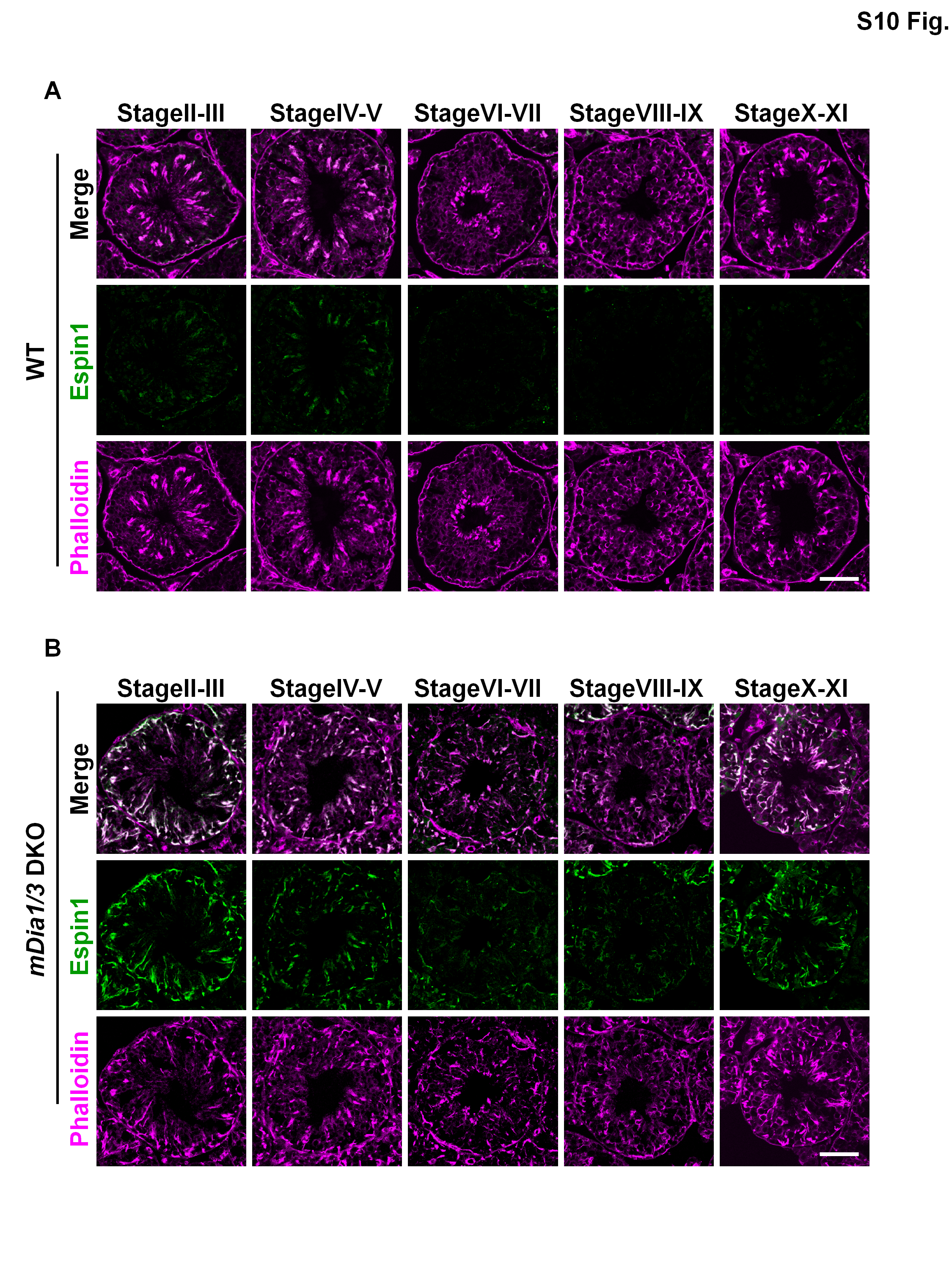

Supplement: S10 Fig — (A) Immunohistochemistry staining for espin1 (green) and phalloidin staining (magenta) of testis sections from WT mice. Espin1 signals were observed at the apical ES junction of elongated spermatid from stages II–V seminiferous tubules of the WT mice. (B) Immunohistochemistry staining for espin1 (green) and phalloidin staining (magenta) of testis sections from mDia1/3 DKO mice. Espin1 signals were ectopically localized to the abnormal F-actin bundles in the process of Sertoli cells throughout the spermatogenic cycle. Scale bars, 100 μm. DKO, double knockout; ES, ectoplasmic specialization; F-actin, filamentous actin; mDia1/3, mammalian diaphanous homolog1/3; WT, wild-type. (TIF) [file pbio.2004874.s010.tif]

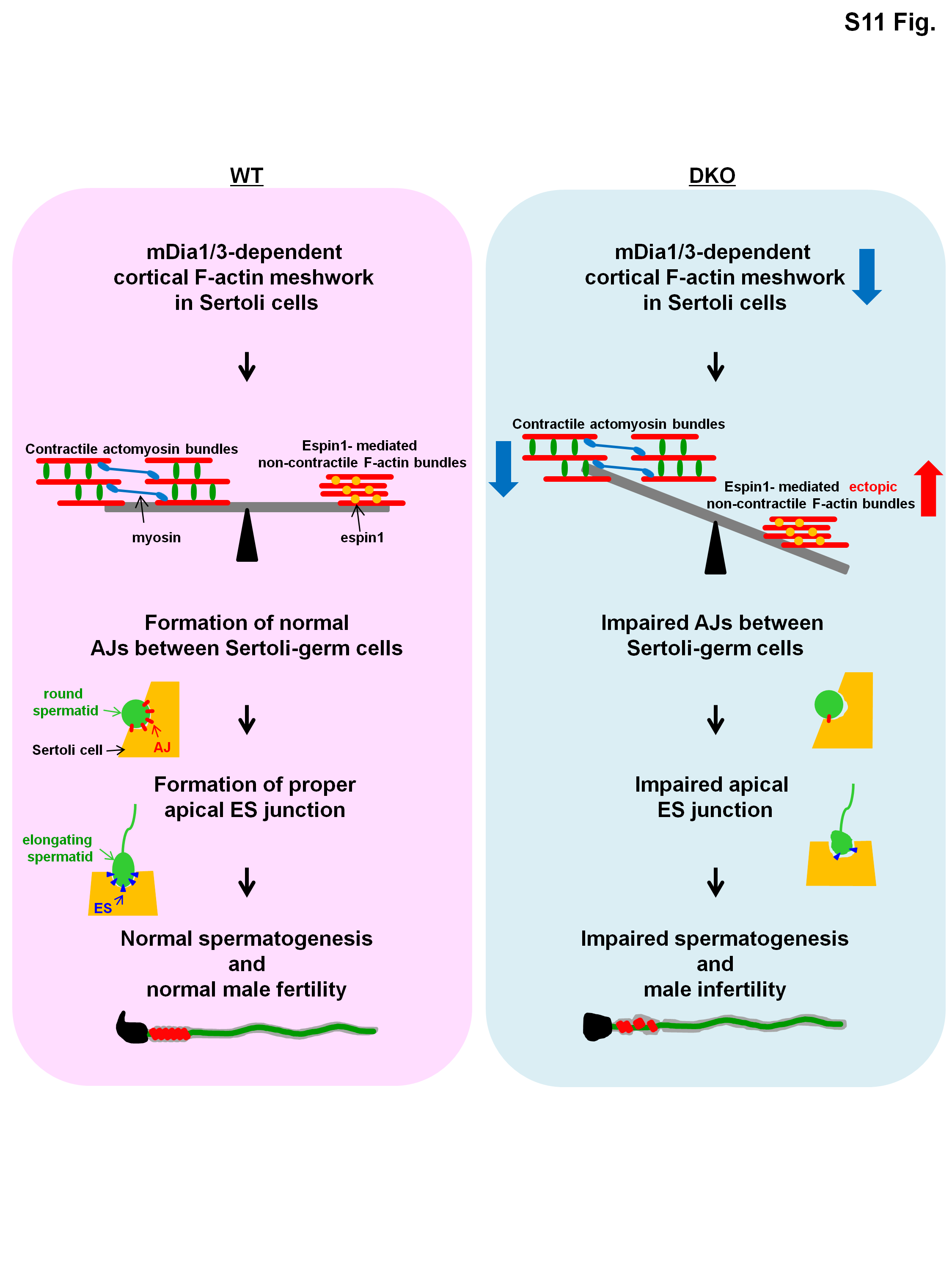

Supplement: S11 Fig — F-actin, filamentous actin; mDia1/3, mammalian diaphanous homolog1/3. (TIF) [file pbio.2004874.s011.tif]
